# Multivariate polynomial fit: Decay heat removal system and pectin degrading Fe_3_O_4_‐SiO_2_ nanobiocatalyst activity

**DOI:** 10.1049/nbt2.12034

**Published:** 2021-04-20

**Authors:** Boopathi Muthusamy, Sujatha Ramalingam, Senthil Kumar Chandran, Sathish Kumar Kannaiyan

**Affiliations:** ^1^ Department of Mathematics Sri Sivasubramaniya Nadar College of Engineering Kalavakkam Kancheepuram India; ^2^ Southern Regional Regulatory Centre Atomic Energy Regulatory Board Chennai India; ^3^ Department of Chemical Engineering Sri Sivasubramaniya Nadar College of Engineering Kalavakkam Kancheepuram India

## Abstract

Herein, multivariate Lagrange's interpolation polynomial (MLIP) and multivariate least square (MLS) methods are used to derive linear and higher‐order polynomials for two varied applications. (1) For an effective fabrication of Pectin degrading Fe_3_O_4_‐SiO_2_ Nanobiocatalyst activity (IU/mg). Here, the three parameters namely: pH value, pectinase loading and temperature as independent variables are optimized for the maximal of anobiocatalyst activity as a dependent variable. (2) For a passive system reliability estimation of decay heat removal (DHR) of a nuclear power plant. The success criteria of the system depend on three types temperature that do not exceed their respective design safety limits and are considered as dependent variables and 14 significant parameters were used as independent variables. Statistically, the validation of these multivariate polynomials are done by testing of hypothesis. Comparative study of the proposed approach gives significance results in the first application have the optimum conditions for maximum activity using linear MLIP method is: 58.64 with pH = 4, pL = 250 and Temp = 4°C. The maximum activity using second order MLIP method is 59.825 and method of MLS is 59.8249 with the optimized values of an independent variables pH = 4, pL = 300 and Temp = 8°C depicted in Table 1. In DHR system, the significance results are obtained and depicted in Table 2.

## 1 INTRODUCTION

Interpolation is one of the most important technique in numerical analysis for the solutions of many real‐time

applications in science and engineering [1]. It is frequently used to approximate the polynomials for a given set of data. In most of the cases, an unique analytical expression can be derived by interpolation of simple algebraic expressions such as: polynomials, exponential, logarithmic functions and power curves which enable us to represent a relationship between dependent over independent variables [2]. However, for huge data, derivation of interpolating polynomial is very tedious and more time consuming. Alternatively, the regression analysis is used to fit a relationship between the simulated values to the actual data set. Clearly, it expresses a polynomial representation depending on the existence of a relationship among various independent variables. In some cases, the observed data are imprecise while using the uncertainty theory to design the uncertain regression model of these observed data as uncertain variables [3]. Also, estimating the parameters of uncertain regression model using principal of least square. Usually, the least square method is used in linear regression for a best fit of a line or curve. This method calculates to minimize the sum of the squares of offsets for pairs of data points from a curve. Squares of these offsets are called residuals [4, 5].

For any simulation study in science and engineering, it is not always feasible to develop an exact representation of input and output response. Also, if the problem is of higher dimension and involves large amount of data, validation becomes difficult. Hence, an universally acceptable method for a real‐time application is never possible [2]. In such a case, the linear regression is used for fitting as a special kind of multiple linear regression models. This is a popular statistical technique that can be widely used to derive the linear relationship between variables. Most frequently, the practical data have non‐linear or curvilinear function representation the polynomial regressions methods are appropriate. Also, the parameters of the polynomial regression are determined by method of least square. This method having a problem of computing the correlation coefficient among variables of the polynomial regression that possess an ill‐conditioned/multi‐collinearity. It shows the computations of matrix operations in least square method approaches to singular value. Thus, the multi‐collinearity possess an unreliable of the parameter estimation in the polynomial regression over the large variances [6, 7]. There are many steps focused to fit better regression models to understand the relationship between variables of the data set, deriving relationship using prediction models, error estimation and inferences about results of the prediction model to fit actual data set and this will helps to determine the correlation between the variables. Various regression analysis are used for data fitting like: linear and multivariate linear regressions, polynomial and multivariate polynomial regressions, and quadratic polynomial regressions etc. [8]. The other methods like, Response Surface Methodology (RSM), central composite design experiment are used to fit a mathematical expression between dependent over independent variables [9]. Generally, the multivariate polynomial regression is extended from the simple regression model [10]. The disadvantages of the multivariate regression models does not have scope for the smaller data set with high‐levels of mathematical computations. Sometimes, the output of the model cannot be easily interpreted and the error outputs are not identical. Reasonably for this situation, most of the researchers preferred method of least square and method of least absolute value to fit best curve fit. Also, to avoid ill‐conditioned situation the multivariate Lagrange's interpolating polynomial (MLIP) method is preferred as in view of its simplistic approach [11], with error approaches to zero.

The objective of the present study is to fit a linear and higher‐order polynomials by MLIP method and method of multivariate least square (MLS) [10] for real‐time applications. Statistically, the validation of derived polynomials by Chi‐square testing for goodness of fit is applied [12], and the validation of huge number of polynomials follows large sample test.

Herein, Section 2 discusses the related works. Schematic representation with advantages of the proposed approach are explained in Section 3. Section 4 discusses the MLIP method and method of MLS is presented in Section 5. Proposed approach is applied for two real‐time applications, the statistical validations with comparative results are discussed in Section 6. Short summary of Matlab computations with statistical validations are presented in Section 7 and Section 8 respectively. Finally, the results and conclusions are summarized in Section 9.

## 2 RELATED WORKS

Many of the real‐world applications have multiple input parameters and output responses. In such applications, multivariate polynomial is derived for *m* input variables with degree *p* over *p* + *m* combinations of *p* distinct data points [13]. These methods are mathematically discussed using set of input arguments in [14], the derived polynomials by rational numbers [15] and Newton form of multivariate polynomial interpolation are discussed in [2]. Regression model is more useful for predicting the forecasting technique of future values, data mining and many applications in engineering domains. In addition, the Bayesian latent factor regression for multivariate functional data with variable selection in [16]. In chemical engineering application, the optimization of acid dye bio‐sorption of brewery waste biomass using RSM is considered. The percentage of removal efficiency is significantly influenced by time, pH, adsorbent dosage with initial dye concentration and multivariate polynomial methods are adopted subsequently [17]. One‐Factor At‐a‐Time (OFAT) procedure is followed by RSM with canonical analysis was used to optimize the immobilization parameters such as: glutaraldehyde concentration, pH value, temperature and pectinase loading for the effective fabrication characterization and application of pectin degrading Fe_3_O_4_‐SiO_2_ nanobiocatalyst were discussed in [9, 18]. The other similar real‐world application where multivariate polynomial is used as characterization of the relationship between strains and drilling depth in a metallurgical plant is discussed in [19]. Similarly, for the estimation of reliability of a passive decay heat removal system of a nuclear power plant (NPP), one simple approach to avoid rigorous computational exercise is by adopting RSM [20]. Also, the regression analysis and curve fitting models are used to approximate the analytic expression of given discrete data. However, the linear and non‐linear regression models are widely used for passive system reliability estimation [21]. Multivariate polynomial fit based on the RSM produces reasonably realistic estimation. The results are not only computationally efficient but also consistent. The accuracy and efficiency of this approach is compared to the direct Monte–Carlo simulation [22]. The success criteria of the system is taken from [23] and the failure probability evaluation of the passive system analysis is verified with other techniques such as fuzzy Monte–Carlo simulation [24]. Passive systems involves in new generation nuclear design for safety‐critical functions mainly concentrate to improve safety and reduce human errors with low cost as well as avoid dependence on the external power source. Moreover, the reliability estimation of passive system is theoretically more reliable as compared to active components. But, uncertainty involved in this system to be very large. Therefore, we necessarily need to quantify the uncertainty. Some uncertainties may happen due to insufficient information, such a case the fuzzy set theory is more appropriate instead of probability concepts [21]. For instance, to handle large amount of data with finite number of thermal‐hydraulic code runs, the regression analysis was performed. To perform an effective regression models, it is necessary to find the effect of every input parameter that goes to into the system [25].

### 2.1 Scope of the proposed study

From the above detailed review, the proposed study addresses the research gap for polynomial contributions in real‐time applications.

1. To develop a relationship between several independent and dependent variables,
2. Multivariate mathematical methods are preferred,
3. To validate the multivariate polynomials,
4. Testing of hypothesis is applied,
5. Different real‐time applications are discussed,
6. Comparative study of the proposed approach.

## 3 SCHEMATIC REPRESENTATION OF THE PROPOSED APPROACH

This section discusses the detailed workflow, proposed approach, merits and demerits of the mathematical models for real‐time applications.

### 3.1 Context and solution technique

In this section, the aim of the study, mathematical methods, procedure for deriving polynomials and validations are discussed in detail.

*Aims:* The advantages of multivariate polynomial methods to develop the relationship between dependent and several independent variables which will be used to identify the significant contributor of the output in an application.

**FIGURE 1:**
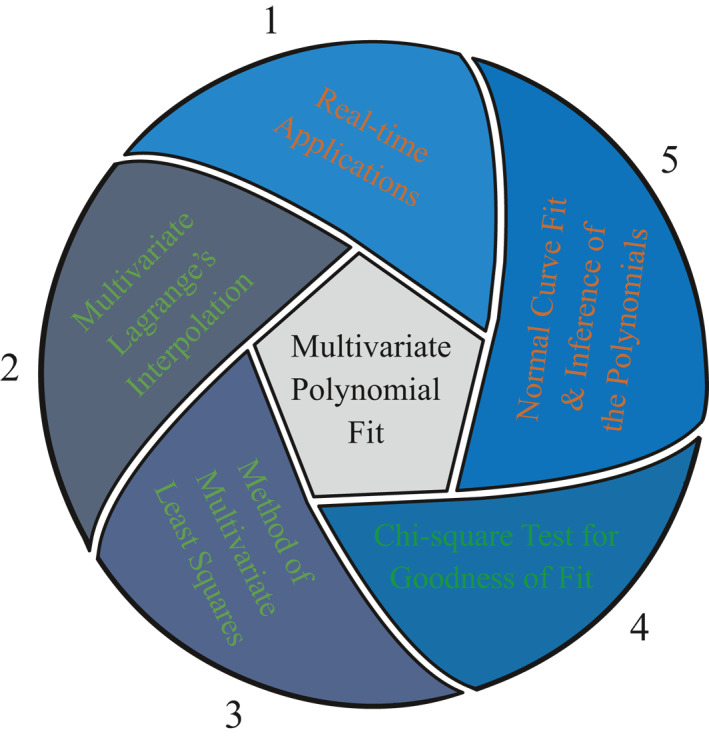
Factors considered for fitting of multivariate polynomials by mathematical methods

*Methods:* For the above context, the mathematical methods are very much essential for solving real‐time applications. Therefore, MLIP and method of MLS are preferred to fit a linear and higher‐order polynomials for the above real‐time applications. The necessary and sufficient condition for the uniqueness of MLIP method is that the sample square matrix is non‐singular [13, 26]. In practical application, if the sample matrix is singular then the MLIP method fails (ill‐conditioned). In such a case, the method of MLS is preferred to quantify the polynomial coefficients [4]. Here, Microsoft Mathematics tool and Matlab programming language were used for performing high‐level computations of this approach. Thus, the list of factors are ordered and is used to fit a multivariate polynomial shown in Figure 1.

**FIGURE 2:**
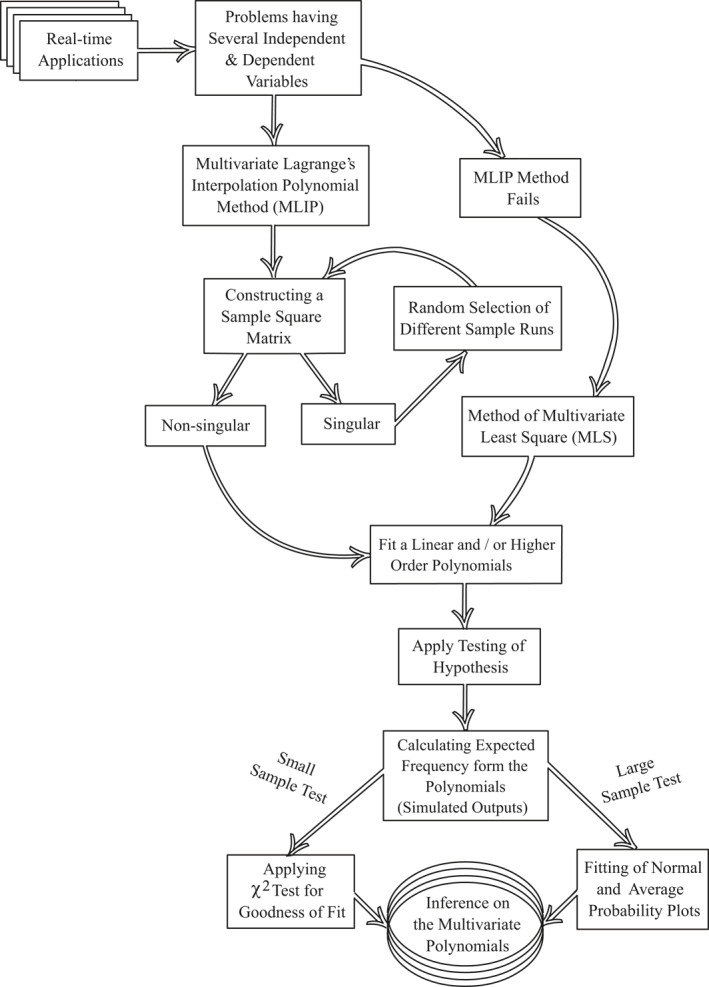
Detailed workflow for fitting of multivariate polynomials

*Procedure:* A detailed procedure for deriving multivariate polynomials with their statistical validation of the applications are shown in Figure 2. The results were carried out and we concentrated more on stress in the statistical validation to assess the practical significance of the problem.

*Polynomial Validation:* To verify and validate the multivariate linear and/or higher‐order polynomials by testing of hypothesis is preferred. Moreover, the contribution of the statistical analysis is to assess the practical significance of the testing results. Thus, the derivation of multivariate polynomials depends on each sample square matrix of the above methods. More clearly, the sample square matrix is selected based on the size of sample runs. The above mathematical methods are directly applied for one and only one sample matrix to derive polynomial which is possible only when the number of independent variables involved in the practical application is same as the number of sample runs (each non‐singular matrix of the method derives one multivariate polynomial). Otherwise, if the size of sample runs is more as compared to number of independent variables, then more than one polynomial computation is applicable for the simulation study. Therefore, the above methods are applicable for polynomial computations while the selection of sample square matrices depending on the combination among all sample runs, that is the size of the sample runs *N* is more as compared to number of variables *K* (selection of *K* multivariate vectors out of sample size *N*) then counting the selection of sample square matrices is

NK=N!K!(N−K)!,K=0,1,2,3…,N

Finally, validation of the derived polynomial follows small sample test, if the number of sample square matrices are less or equal to 30. Otherwise, the method follows large sample test. In small sample test, the *χ* ^2^ testing for goodness of fit is performed in which the experimental outputs are taken as observed frequency (*O*_*i*_). The simulated outputs are the expected frequencies (*E*_*i*_) calculated from derived polynomials by both mathematical methods which are treated as output arguments depending on the corresponding input data set. To validate these polynomials, the following hypothetical assumptions are:

1. Testing the hypothesis against the derived polynomials of the application which depends on the number of matrices (size say: *n*). Also, the null hypothesis of the problem is decided based on the sample square matrices which are less than or equal to 30.
2. Under *H*_0_: ‘*The multivariate polynomial fit is good one*’. That is, there is no significant difference between the observed and expected frequencies.
3. Otherwise *H*_1_: ‘*The multivariate polynomial fit is not good one’*. That is, there is a significant difference between the observed and expected frequencies.
4. *Test statistic:* Under *H*_0_, the test statistic is given by
$$\chi^{2}=\overset{k}{\underset{i=1}{\sum }}\frac{{\left(O_{i}-E_{i}\right)}^{2}}{E_{i}}∼\left(k-1\right)\;\;\;d.\;f$$
5. *Inferences:* Let $\chi_{\alpha }^{2}$ be the table value of Chi‐square distribution at *α* level of significance with (*k* − 1) d.f. If the hypothesis is accepted, then the polynomial fit is a true representation of the problem under study. Otherwise, it is concluded that the fit is not a true representation of the application.
6. If the number of matrices are greater than 30 then to validate the above null hypothesis under large sample test.

*Case Study:* There are two different applications were discussed below.

1. Application of multivariate polynomial for estimation of reliability for a safety‐critical system in an NPP.
2. An effective fabrication of pectin degrading Fe_3_O_4_‐SiO_2_ Nanobiocatalyst activity (IU/mg).

**FIGURE 3:**
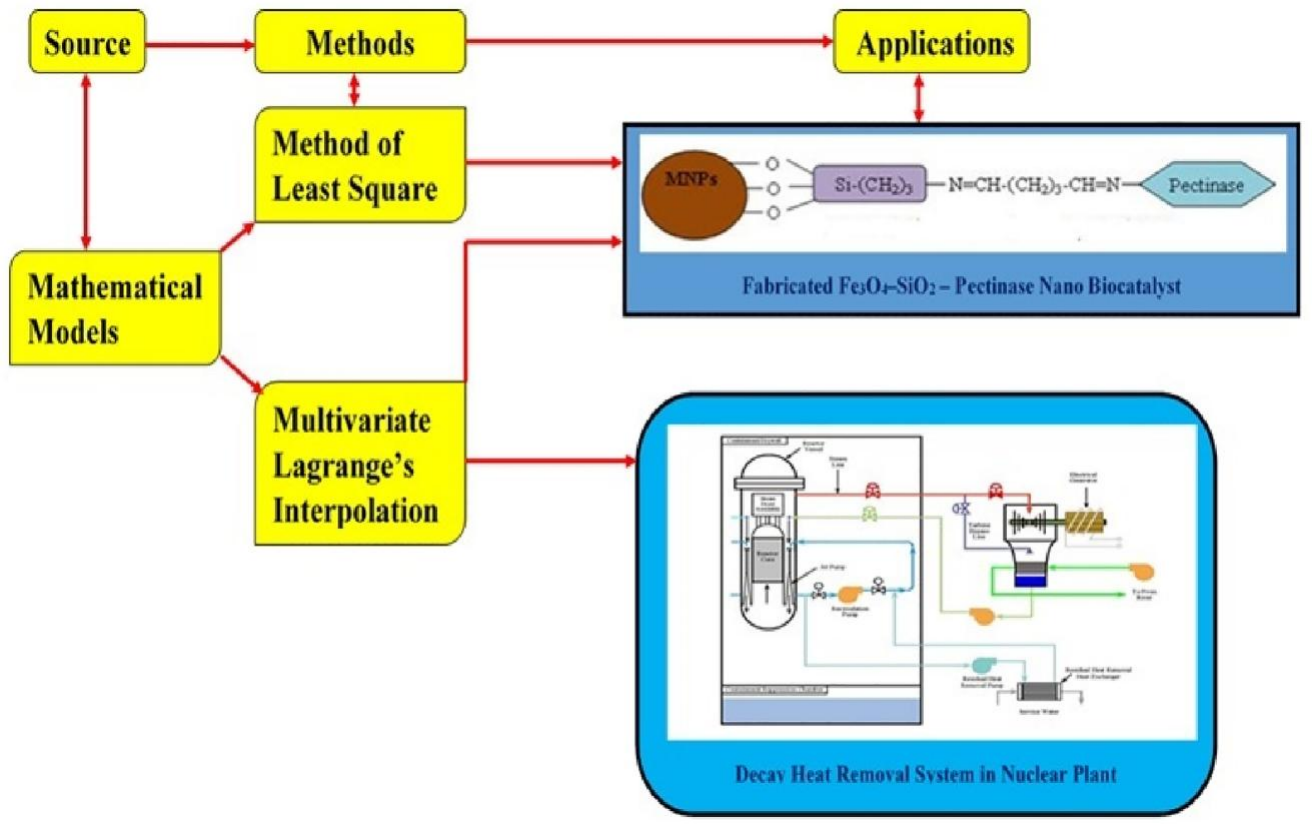
Multivariate polynomial fit: real‐time applications

The mathematical approach for the solutions of above case study are shown in Figure 3.

### 3.2 Merits and De‐merits: multivariate polynomial methods

The next section outlines the merits and demerits of the mathematical methods in practical situations.

#### 3.2.1 Merits

The merits of the proposed work are discussed in the following.

1. Mathematical relationship between variables response is developed as a multivariate linear and higher‐order polynomials using MLIP and/or MLS methods.
2. Extrapolating beyond the range of input arguments, the input and output relationship is developed without repeating the experiments (simulation).
3. The polynomial representation is quickly solvable and consumes less time to obtain the desired results.
4. The verification and validation of the polynomials can be carried out using *χ* ^2^ testing for goodness of fit. Otherwise, it follows large sample test.
5. The multivariate polynomial methods are more helpful for further reliability quantifications in safety‐critical applications.

#### 3.2.2 Demerits

The demerits of the method is given below.

1. When the application consists of huge number of parameters, it is very tedious to develop an exact relationship between input and output arguments.
2. The computational complexity increases with the number of variables making tool‐based polynomial computation difficult for method of MLS.
3. Derivation of second‐order polynomials is difficult; since the method of MLS does not guarantee if the determinant of sample square matrices are zero or tends to zero (ill‐conditioned criterion).

The unique multivariate polynomial fit derived by MLIP method is discussed in the next section.

## 4 MULTIVARIATE LAGRANGE's INTERPOLATING POLYNOMIAL

Let *f*(*X*_1_, *X*_2_, *…*, *X*_*m*_) be multinomial function of *m* independent variables *x*_1_, *x*_2_, …, *x*_*m*_ over degree *p* in [27]. There are (*p* + *m*)*C*_*p*_ = *ρ* as number of terms in *f*, the necessary condition that *ρ* number of distinct data points *f*(*x*_1,*i*_, *x*_2,*i*_, *…*, *x*_*m*,*i*_) = *f*_*i*_ in $R^{m+1}$ and *f* is uniquely defined [8, 10]. Then,

(1) $$f\left(X_{1},\ldots ,X_{m}\right)=\underset{e_{i}.\;1\leq p}{\sum }\alpha_{e_{i}}\;X^{e_{i}},\;\;\;\;\;\;\;\;1\leq i\leq \rho$$

Where $\alpha_{e_{i}}$ are the coefficients in *f*. The MLIP function is of the form

(2) $$L\left(x_{i}\right)=\overset{\rho }{\underset{i=1}{\sum }}f_{i}\;l_{i}\left(x\right)$$

Here, *l*_*i*_(*X*) is a multinomial function of several independent variables *X*_1_, *X*_2_, …, *X*_*m*_ having the property that *X* is equal to *i* ^*th*^ data point, that is *X* = *x*_*i*_ (*x*_1,*i*_, *x*_2,*i*_, …, *x*_*m*,*i*_). Moreover, *l*_*i*_(*X*) satisfies the following condition:

(3) $$l_{i}\left(X\right)=\left\{\begin{array}{cc} l_{i}\left(x_{i}\right)=1,\; & \text{if}\;j=i;\\ l_{j}\left(x_{i}\right)=0,\; & \text{if}\;j\neq i. \end{array}\right.$$

The function *f*_*i*_ gives system of linear equation

(4) $$f_{i}=\underset{e_{i}.\;1\leq p}{\sum }\alpha_{e_{i}}\;x_{i}^{e_{i}},\;\;\;\;\;\;\;\;1\leq i\leq \rho$$

The sample square matrix $M=\left(x_{i}^{e_{j}}\right)$ with determinant det(*M*) is non‐singular. Therefore,

(5) $$M_{j}\left(X\right)=\left(\begin{array}{ccc} x_{1}^{e_{1}} & \ldots & x_{1}^{e_{\rho }}\\ \vdots & \cdots & \vdots \\ x_{i}^{e_{1}} & \cdots & x_{i}^{e_{\rho }}\\ \vdots & \cdots & \vdots \\ x_{\rho }^{e_{1}} & \cdots & x_{\rho }^{e_{\rho }} \end{array}\right)\;⟵j^{th}\;row$$

At *X* = *x*_*i*_ in *M*_*j*_(*X*) has *i* ^*th*^ row appears twice, then $det\left({\left(M_{j}\left(X\right)\right)}_{i}\right)=0\;for\;\left(i\neq j\right)$. Hence, $l_{i}\left(X\right)=\frac{\Delta_{i}\left(X\right)}{\Delta }$. The unique multivariate linear and higher‐order polynomial is

(6) $$f\left(X_{1},X_{2},\ldots ,X_{m}\right)=\overset{\rho }{\underset{i=1}{\sum }}\frac{\Delta_{i}\left(X\right)}{\Delta }\times f_{i}$$

If MLIP method fails, then the method of MLS is preferred.

## 5 METHOD OF MULTIVARIATE LEAST SQUARE

The method of MLS for second‐order polynomial over three independent variables has the form

(7) $$t^{\prime }=t_{f}$$

Consider *t*_*f*_ = *a*′ + *b*′*x* + *c*′*y* + *d*′*z* + *e*′*xy* + *f*′*yz* + *g*′*zx* + *h*′*x* ^2^ + *k*′*y* ^2^ + *m*′*z* ^2^. Estimation of the polynomial coefficients *a*′, *b*′, *c*′, *d*′, *e*′, *f*′, *g*′, *h*′, *k*′, *m*′ by applying method of MLS to minimize error *E*_mls_ from the actual *k* data points.

(8) $$E_{\text{mls}}=\overset{k}{\underset{i=1}{\sum }}{\left[t_{i}^{\prime }-t_{f}\right]}^{2}$$

Take partial derivatives of *E*_mls_ in Equation (8) with respect to *a*′, *b*′, *c*′, *d*′, *e*′, *f*′, *g*′, *h*′, *k*′, *m*′ and equating to zero have the following equations:

The set of equations (9) to (18) can be rearranged into matrix form by

(9) $$\begin{array}{c} \overset{k}{\underset{i=1}{\sum }}t_{i}=na^{\prime }+b^{\prime }\overset{k}{\underset{i=1}{\sum }}x_{i}+c^{\prime }\overset{k}{\underset{i=1}{\sum }}y_{i}+d^{\prime }\overset{k}{\underset{i=1}{\sum }}z_{i}+e^{\prime }\overset{k}{\underset{i=1}{\sum }}x_{i}y_{i}+f^{\prime }\overset{k}{\underset{i=1}{\sum }}y_{i}z_{i}\\ +g^{\prime }\overset{k}{\underset{i=1}{\sum }}z_{i}x_{i}+h^{\prime }\overset{k}{\underset{i=1}{\sum }}x_{i}^{2}+k^{\prime }\overset{k}{\underset{i=1}{\sum }}y_{i}^{2}+m^{\prime }\overset{k}{\underset{i=1}{\sum }}z_{i}^{2} \end{array}$$

(10) $$\begin{array}{c} \overset{k}{\underset{i=1}{\sum }}x_{i}t_{i}=a^{\prime }\overset{k}{\underset{i=1}{\sum }}x_{i}+b^{\prime }\overset{k}{\underset{i=1}{\sum }}x_{i}^{2}+c^{\prime }\overset{k}{\underset{i=1}{\sum }}x_{i}y_{i}+d^{\prime }\overset{k}{\underset{i=1}{\sum }}z_{i}x_{i}+e^{\prime }\overset{k}{\underset{i=1}{\sum }}x_{i}^{2}y_{i}+f^{\prime }\overset{k}{\underset{i=1}{\sum }}x_{i}y_{i}z_{i}\\ +g^{\prime }\overset{k}{\underset{i=1}{\sum }}z_{i}x_{i}^{2}+h^{\prime }\overset{k}{\underset{i=1}{\sum }}x_{i}^{3}+k^{\prime }\overset{k}{\underset{i=1}{\sum }}x_{i}y_{i}^{2}+m^{\prime }\overset{k}{\underset{i=1}{\sum }}x_{i}z_{i}^{2} \end{array}$$

(11) $$\begin{array}{c} \overset{k}{\underset{i=1}{\sum }}y_{i}t_{i}=a^{\prime }\overset{k}{\underset{i=1}{\sum }}y_{i}+b^{\prime }\overset{k}{\underset{i=1}{\sum }}x_{i}y_{i}+c^{\prime }\overset{k}{\underset{i=1}{\sum }}y_{i}^{2}+d^{\prime }\overset{k}{\underset{i=1}{\sum }}z_{i}y_{i}+e^{\prime }\overset{k}{\underset{i=1}{\sum }}x_{i}y_{i}^{2}+f^{\prime }\overset{k}{\underset{i=1}{\sum }}y_{i}^{2}z_{i}\\ +g^{\prime }\overset{k}{\underset{i=1}{\sum }}x_{i}y_{i}z_{i}+h^{\prime }\overset{k}{\underset{i=1}{\sum }}x_{i}^{2}y_{i}+k^{\prime }\overset{k}{\underset{i=1}{\sum }}y_{i}^{3}+m^{\prime }\overset{k}{\underset{i=1}{\sum }}y_{i}z_{i}^{2} \end{array}$$

(12) $$\begin{array}{cc} \overset{k}{\underset{i=1}{\sum }}z_{i}t_{i} & =a^{\prime }\overset{k}{\underset{i=1}{\sum }}z_{i}+b^{\prime }\overset{k}{\underset{i=1}{\sum }}x_{i}z_{i}+c^{\prime }\overset{k}{\underset{i=1}{\sum }}y_{i}z_{i}+d^{\prime }\overset{k}{\underset{i=1}{\sum }}z_{i}^{2}+e^{\prime }\overset{k}{\underset{i=1}{\sum }}x_{i}y_{i}z_{i}+f^{\prime }\overset{k}{\underset{i=1}{\sum }}y_{i}z_{i}^{2}\\ +g^{\prime }\overset{k}{\underset{i=1}{\sum }}x_{i}z_{i}^{2}+h^{\prime }\overset{k}{\underset{i=1}{\sum }}x_{i}^{2}z_{i}+k^{\prime }\overset{k}{\underset{i=1}{\sum }}y_{i}^{2}z_{i}+m^{\prime }\overset{k}{\underset{i=1}{\sum }}z_{i}^{3} \end{array}$$

(13) $$\begin{array}{c} \overset{k}{\underset{i=1}{\sum }}x_{i}y_{i}t_{i}=a^{\prime }\overset{k}{\underset{i=1}{\sum }}x_{i}y_{i}+b^{\prime }\overset{k}{\underset{i=1}{\sum }}x_{i}^{2}y_{i}+c^{\prime }\overset{k}{\underset{i=1}{\sum }}x_{i}y_{i}^{2}+d^{\prime }\overset{k}{\underset{i=1}{\sum }}x_{i}y_{i}z_{i}+e^{\prime }\overset{k}{\underset{i=1}{\sum }}x_{i}^{2}y_{i}^{2}\\ +f^{\prime }\overset{k}{\underset{i=1}{\sum }}x_{i}y_{i}^{2}z_{i}+g^{\prime }\overset{k}{\underset{i=1}{\sum }}x_{i}^{2}y_{i}z_{i}+h^{\prime }\overset{k}{\underset{i=1}{\sum }}x_{i}^{3}y_{i}z_{i}+k^{\prime }\overset{k}{\underset{i=1}{\sum }}y_{i}^{3}x_{i}+m^{\prime }\overset{k}{\underset{i=1}{\sum }}x_{i}y_{i}z_{i}^{2} \end{array}$$

(14) $$\begin{array}{c} \overset{k}{\underset{i=1}{\sum }}y_{i}z_{i}t_{i}=a^{\prime }\overset{k}{\underset{i=1}{\sum }}y_{i}z_{i}+b^{\prime }\overset{k}{\underset{i=1}{\sum }}x_{i}y_{i}z_{i}+c^{\prime }\overset{k}{\underset{i=1}{\sum }}y_{i}^{2}z_{i}+d^{\prime }\overset{k}{\underset{i=1}{\sum }}y_{i}z_{i}^{2}+e^{\prime }\overset{k}{\underset{i=1}{\sum }}x_{i}y_{i}^{2}z_{i}\\ +f^{\prime }\overset{k}{\underset{i=1}{\sum }}x_{i}y_{i}^{2}z_{i}^{2}+g^{\prime }\overset{k}{\underset{i=1}{\sum }}x_{i}y_{i}z_{i}^{2}+h^{\prime }\overset{k}{\underset{i=1}{\sum }}x_{i}^{2}y_{i}z_{i}+k^{\prime }\overset{k}{\underset{i=1}{\sum }}y_{i}^{3}z_{i}+m^{\prime }\overset{k}{\underset{i=1}{\sum }}y_{i}z_{i}^{3} \end{array}$$

(15) $$\begin{array}{c} \overset{k}{\underset{i=1}{\sum }}x_{i}z_{i}t_{i}=a^{\prime }\overset{k}{\underset{i=1}{\sum }}x_{i}z_{i}+b^{\prime }\overset{k}{\underset{i=1}{\sum }}x_{i}^{2}z_{i}+c^{\prime }\overset{k}{\underset{i=1}{\sum }}x_{i}y_{i}z_{i}+d^{\prime }\overset{k}{\underset{i=1}{\sum }}x_{i}z_{i}^{2}+e^{\prime }\overset{k}{\underset{i=1}{\sum }}x_{i}^{2}y_{i}z_{i}\\ +f^{\prime }\overset{k}{\underset{i=1}{\sum }}x_{i}y_{i}z_{i}^{2}+g^{\prime }\overset{k}{\underset{i=1}{\sum }}x_{i}^{2}z_{i}^{2}+h^{\prime }\overset{k}{\underset{i=1}{\sum }}x_{i}^{3}z_{i}+k^{\prime }\overset{k}{\underset{i=1}{\sum }}x_{i}y_{i}^{2}z_{i}+m^{\prime }\overset{k}{\underset{i=1}{\sum }}x_{i}z_{i}^{3} \end{array}$$

(16) $$\begin{array}{c} \overset{k}{\underset{i=1}{\sum }}x_{i}^{2}t_{i}=a^{\prime }\overset{k}{\underset{i=1}{\sum }}x_{i}^{2}+b^{\prime }\overset{k}{\underset{i=1}{\sum }}x_{i}^{3}+c^{\prime }\overset{k}{\underset{i=1}{\sum }}x_{i}^{2}y_{i}+d^{\prime }\overset{k}{\underset{i=1}{\sum }}x_{i}^{2}z_{i}+e^{\prime }\overset{k}{\underset{i=1}{\sum }}x_{i}^{3}y_{i}+f^{\prime }\overset{k}{\underset{i=1}{\sum }}x_{i}^{2}y_{i}z_{i}\\ +g^{\prime }\overset{k}{\underset{i=1}{\sum }}x_{i}^{3}z_{i}+h^{\prime }\overset{k}{\underset{i=1}{\sum }}x_{i}^{4}+k^{\prime }\overset{k}{\underset{i=1}{\sum }}x_{i}^{2}y_{i}^{2}+m^{\prime }\overset{k}{\underset{i=1}{\sum }}x_{i}^{2}z_{i}^{2} \end{array}$$

(17) $$\begin{array}{c} \overset{k}{\underset{i=1}{\sum }}y_{i}^{2}t_{i}=a^{\prime }\overset{k}{\underset{i=1}{\sum }}y_{i}^{2}+b^{\prime }\overset{k}{\underset{i=1}{\sum }}x_{i}y_{i}^{2}+c^{\prime }\overset{k}{\underset{i=1}{\sum }}y_{i}^{3}+d^{\prime }\overset{k}{\underset{i=1}{\sum }}y_{i}^{2}z_{i}+e^{\prime }\overset{k}{\underset{i=1}{\sum }}x_{i}y_{i}^{3}\\ +f^{\prime }\overset{k}{\underset{i=1}{\sum }}y_{i}^{3}z_{i}+g^{\prime }\overset{k}{\underset{i=1}{\sum }}x_{i}y_{i}^{2}z_{i}+h^{\prime }\overset{k}{\underset{i=1}{\sum }}x_{i}^{2}y_{i}^{2}+k^{\prime }\overset{k}{\underset{i=1}{\sum }}y_{i}^{4}+m^{\prime }\overset{k}{\underset{i=1}{\sum }}y_{i}^{2}z_{i}^{2} \end{array}$$

(18) $$\begin{array}{c} \overset{k}{\underset{i=1}{\sum }}z_{i}^{2}t_{i}=a^{\prime }\overset{k}{\underset{i=1}{\sum }}z_{i}^{2}+b^{\prime }\overset{k}{\underset{i=1}{\sum }}x_{i}z_{i}^{2}+c^{\prime }\overset{k}{\underset{i=1}{\sum }}y_{i}z_{i}^{2}+d^{\prime }\overset{k}{\underset{i=1}{\sum }}z_{i}^{3}+e^{\prime }\overset{k}{\underset{i=1}{\sum }}x_{i}y_{i}z_{i}^{2}\\ +f^{\prime }\overset{k}{\underset{i=1}{\sum }}y_{i}z_{i}^{3}+g^{\prime }\overset{k}{\underset{i=1}{\sum }}x_{i}z_{i}^{3}+h^{\prime }\overset{k}{\underset{i=1}{\sum }}x_{i}^{2}z_{i}^{2}+k^{\prime }\overset{k}{\underset{i=1}{\sum }}y_{i}^{2}z_{i}^{2}+m^{\prime }\overset{k}{\underset{i=1}{\sum }}z_{i}^{4} \end{array}$$

(19) $$\begin{array}{ll} & \overset{k}{\underset{i=1}{\sum }}\left(\;\begin{array}{cccccccccc} n & x_{i} & y_{i} & z_{i} & x_{i}y_{i} & y_{i}z_{i} & z_{i}x_{i} & x_{i}^{2} & y_{i}^{2} & z_{i}^{2}\\ x_{i} & x_{i}^{2} & x_{i}y_{i} & z_{i}x_{i} & x_{i}^{2}y_{i} & x_{i}y_{i}z_{i} & z_{i}x_{i}^{2} & x_{i}^{3} & x_{i}y_{i}^{2} & x_{i}z_{i}^{2}\\ y_{i} & x_{i}y_{i} & y_{i}^{2} & z_{i}y_{i} & x_{i}y_{i}^{2} & y_{i}^{2}z_{i} & x_{i}y_{i}z_{i} & x_{i}^{2}y_{i} & y_{i}^{3} & y_{i}z_{i}^{2}\\ z_{i} & x_{i}z_{i} & y_{i}z_{i} & z_{i}^{2} & x_{i}y_{i}z_{i} & y_{i}z_{i}^{2} & x_{i}z_{i}^{2} & x_{i}^{2}z_{i} & y_{i}^{2}z_{i} & z_{i}^{3}\\ x_{i}y_{i} & x_{i}^{2}y_{i} & x_{i}y_{i}^{2} & x_{i}y_{i}z_{i} & x_{i}^{2}y_{i}^{2} & x_{i}y_{i}^{2}z_{i} & x_{i}^{2}y_{i}z_{i} & x_{i}^{3}y_{i}z_{i} & y_{i}^{3}x_{i} & x_{i}y_{i}z_{i}^{2}\\ y_{i}z_{i} & x_{i}y_{i}z_{i} & y_{i}^{2}z_{i} & y_{i}z_{i}^{2} & x_{i}y_{i}^{2}z_{i} & y_{i}^{2}z_{i}^{2} & x_{i}y_{i}z_{i}^{2} & x_{i}^{2}y_{i}z_{i} & y_{i}^{3}z_{i} & y_{i}z_{i}^{3}\\ z_{i}x_{i} & x_{i}^{2}z_{i} & x_{i}y_{i}z_{i} & x_{i}z_{i}^{2} & x_{i}^{2}y_{i}z_{i} & x_{i}y_{i}z_{i}^{2} & x_{i}^{2}z_{i}^{2} & x_{i}^{3}z_{i} & x_{i}y_{i}^{2}z_{i} & x_{i}z_{i}^{3}\\ x_{i}^{2} & x_{i}^{3} & x_{i}^{2}y_{i} & x_{i}^{2}z_{i} & x_{i}^{3}y_{i}z_{i} & x_{i}^{2}y_{i}z_{i} & x_{i}^{3}z_{i} & x_{i}^{4} & x_{i}^{2}y_{i}^{2} & x_{i}^{2}z_{i}^{2}\\ y_{i}^{2} & x_{i}y_{i}^{2} & y_{i}^{3} & y_{i}^{2}z_{i} & x_{i}y_{i}^{3} & y_{i}^{3}z_{i} & x_{i}y_{i}^{2}z_{i} & x_{i}^{2}y_{i}^{2} & y_{i}^{4} & y_{i}^{2}z_{i}^{2}\\ z_{i}^{2} & x_{i}z_{i}^{2} & y_{i}z_{i}^{2} & z_{i}^{3} & x_{i}y_{i}z_{i}^{2} & y_{i}z_{i}^{3} & x_{i}z_{i}^{3} & x_{i}^{2}z_{i}^{2} & y_{i}^{2}z_{i}^{2} & z_{i}^{4} \end{array}\;\right)\\ & \;\left(\begin{array}{c} a^{\prime }\\ b^{\prime }\\ c^{\prime }\\ d^{\prime }\\ e^{\prime }\\ f^{\prime }\\ g^{\prime }\\ h^{\prime }\\ k^{\prime }\\ m^{\prime } \end{array}\right)=\overset{k}{\underset{i=1}{\sum }}\left(\begin{array}{c} t_{i}\\ x_{i}t_{i}\\ y_{i}t_{i}\\ z_{i}t_{i}\\ x_{i}y_{i}t_{i}\\ y_{i}z_{i}t_{i}\\ x_{i}z_{i}t_{i}\\ x_{i}^{2}t_{i}\\ y_{i}^{2}t_{i}\\ z_{i}^{2}t_{i} \end{array}\right) \end{array}$$

The above Equation (19), can be written as the Vandermonde square matrix over three independent variables. Then,

(20) $$\begin{array}{cc} \left(\begin{array}{cccccccccc} 1 & x_{1} & y_{1} & z_{1} & x_{1}y_{1} & y_{1}z_{1} & z_{1}x_{1} & x_{1}^{2} & y_{1}^{2} & z_{1}^{2}\\ 1 & x_{2} & y_{2} & z_{2} & x_{2}y_{2} & y_{2}z_{2} & z_{2}x_{2} & x_{2}^{2} & y_{2}^{2} & z_{2}^{2}\\ 1 & x_{3} & y_{3} & z_{3} & x_{3}y_{3} & y_{3}z_{3} & z_{3}x_{3} & x_{3}^{2} & y_{3}^{2} & z_{3}^{2}\\ 1 & x_{4} & y_{4} & z_{4} & x_{4}y_{4} & y_{4}z_{4} & z_{4}x_{4} & x_{4}^{2} & y_{4}^{2} & z_{4}^{2}\\ 1 & x_{5} & y_{5} & z_{5} & x_{5}y_{5} & y_{5}z_{5} & z_{5}x_{5} & x_{5}^{2} & y_{5}^{2} & z_{5}^{2}\\ 1 & x_{6} & y_{6} & z_{6} & x_{6}y_{6} & y_{6}z_{6} & z_{6}x_{6} & x_{6}^{2} & y_{6}^{2} & z_{6}^{2}\\ 1 & x_{7} & y_{7} & z_{7} & x_{7}y_{7} & y_{7}z_{7} & z_{7}x_{7} & x_{7}^{2} & y_{7}^{2} & z_{7}^{2}\\ 1 & x_{8} & y_{8} & z_{8} & x_{8}y_{8} & y_{8}z_{8} & z_{8}x_{8} & x_{8}^{2} & y_{8}^{2} & z_{8}^{2}\\ 1 & x_{9} & y_{9} & z_{9} & x_{9}y_{9} & y_{9}z_{9} & z_{9}x_{9} & x_{9}^{2} & y_{9}^{2} & z_{9}^{2}\\ 1 & x_{10} & y_{10} & z_{10} & x_{10}y_{10} & y_{10}z_{10} & z_{10}x_{10} & x_{10}^{2} & y_{10}^{2} & z_{10}^{2} \end{array}\right)\\ \;\left(\begin{array}{c} a^{\prime }\\ b^{\prime }\\ c^{\prime }\\ d^{\prime }\\ e^{\prime }\\ f^{\prime }\\ g^{\prime }\\ h^{\prime }\\ k^{\prime }\\ m^{\prime } \end{array}\right) & =\left(\begin{array}{c} t_{1}\\ t_{2}\\ t_{3}\\ t_{4}\\ t_{5}\\ t_{6}\\ t_{7}\\ t_{8}\\ t_{9}\\ t_{10} \end{array}\right) \end{array}$$

(21) $$\begin{array}{ccc} Van*A_{\text{mls}} & = & Y \end{array}$$

Now, premultiply by *Van* ^*T*^ on both sides of Equation (21). Then,

$$\left(Van^{T}*Van\right)*A_{\text{mls}}={\left(Van\right)}^{T}*Y$$

Therefore, the co‐efficient of MLS is computed as

(22) $$A_{\text{mls}}={\left(Van^{T}*Van\right)}^{-1}*Van^{T}*Y$$

The matrix *A*_mls_ gives the coefficient of the second‐order multivariate polynomial. This way, MLS can be used to fit a polynomial for real‐time applications with large number of parameters. In the next section, application of both mathematical methods are demonstrated through case studies.

## 6 CASE STUDY

In this section, two different case studies were discussed. The approach for the relationship between case studies and mathematical methods as graphically shown in Figure 3. The detailed workflow for fitting linear and higher‐order multivariate polynomials were shown in Figure 2. Moreover, the solution methodology described by three steps to solve the real time applications are listed in the following order.

1. A brief study and objective of the applications
2. Mathematical analysis (derivation of polynomials)
3. Statistical validation of the polynomials (testing of hypothesis)
4. Comparative study of the proposed approach

**FIGURE 4:**
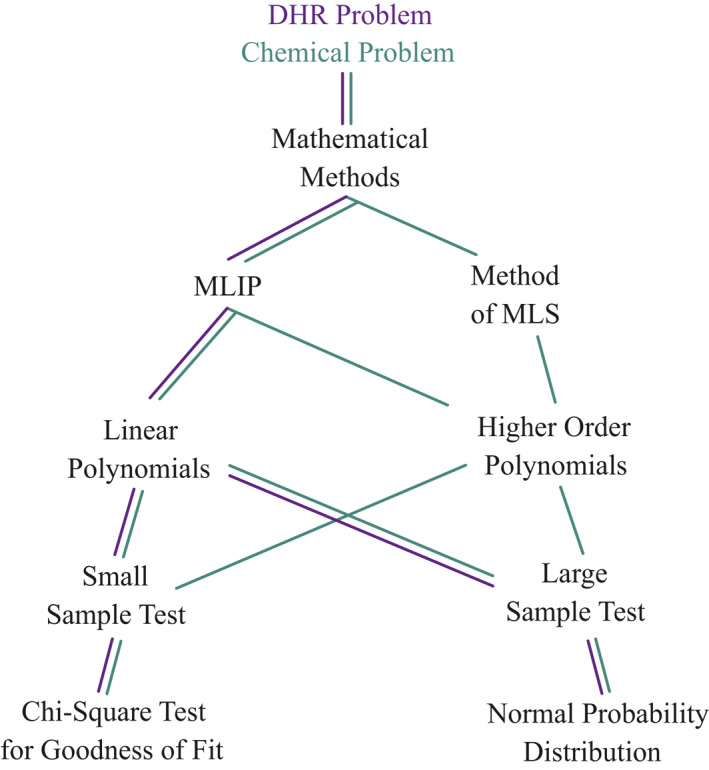
Statistical validation procedure for derived multivariate polynomial models

The statistical validation procedure for the derived multivariate polynomial is shown in Figure 4.

### 6.1 Application 1: an effective fabrication of pectin degrading Fe_3_O_4_‐SiO_2_ nanobiocatalyst activity (IU/mg)

This application belongs to chemical engineering domain and the detailed experiments are discussed in Sections 6.1.1 and 6.1.2. From the detailed description of problem, the proposed study leads further to a mathematical analysis presented in Section 6.1.3 onwards.

#### 6.1.1 Synthesis of ASMPNs and its pectinase immobilization

Magnetic Nano‐Particles (MNPs) was synthesized using FeCl_2_ ⋅ 4H_2_O and FeCl_3_ ⋅ 6H_2_O with molar ratio of 1:2 in deionized water with NH_4_OH as reducing agent. Silica was coated onto the MNPs using detraethoxyortho silicate. Amino (NH2) functional group was imparted using APTES (3‐aminopropyl triethoxysilance). The resulting Silica‐Coated Amino functionalized Magnetic Nano‐Particles (ASMNPs) was activated using glutaraldehyde solutions [18]. The activation was carried out by varying the concentration of glutaraldehyde (0–12)%. 5 mg of activated ASMPs was added to the micro litres of pectinase solution (50, 100, 150, 200, 250, 300 and 350) using acetate and phosphate buffer for various pH (3, 4, 5, 6, 7 and 8) and sonicated for 5 min. The resulting mixture was stored for 1 h at different temperature (0, 4, 8, 12 and 16) for immobilization. The nanoparticles was removed using permanent magnet and washed with clean deionized water.

#### 6.1.2 Activity of Fe_3_O_4_‐SiO_2_ nanobiocatalyst

The activity of pectin degrading Fe_3_O_4_‐SiO_2_ nanobiocatalyst was determined by measuring the reducing sugar produced as a result of reaction between pectinase and pectin. About 500 *μ*L of varying concentrations of pectinase (free pectinase and bound pectinase separately) solution was prepared using 0.1 M acetate buffer and this was added to 1.0 ml of pectin solution (prepared using 0.1 M acetate buffer) containing 2.0 mg of pectin. The reaction mixture was incubated for 1 h at 50°C under shaking condition. The concentration of reducing sugar (galacturonic acid) in the supernatant was estimated by DNS method as described by Miller (1959) using d‐(+)‐galacturonic acid monohydrate as standard [18, 28]. One unit of pectinase activity (IU/mg) is defined as the amount of galacturonic acid produced (mol) per mg of pectinase per min at pH 4.0 and 50°C. The maximum activity of Fe_3_O_4_‐SiO_2_ nanobiocatalyst activity was achieved using 10% glutaraldehyde [9]. All experiments were carried out in triplicates and the mean values were calculated to achieve accuracy.

#### 6.1.3 Mathematical analysis

The aim of the current study is to derive a linear and higher order polynomial by MLIP and method of MLS. For this problem, the One‐Factor At‐a‐Time (OFAT) procedure is followed by RSM and canonical analysis is used to optimize the immobilization parameters. Based on the previous study [9], three parameters were found to be governing and hence these three independent variables namely: pH, pectinase loading and temperature were optimized for their maximal Fe_3_O_4_‐SiO_2_ nanobiocatalyst activity (IU/mg) which is a dependent variable. The levels obtained from OFAT loading for three independent variables were pH (4.0–6.0); pectinase loading (200–300 *μ*g); and temperature (0–8°C). This experimental analysis is more useful for fitting of polynomials by these mathematical methods.

#### 6.1.4 MLIP method for Linear Polynomial Computation

The MLIP method is applied to derive a linear multivariate polynomial as similar to previous case study. The results of 20 runs for three independent variables such as, pH value (pH), pectinase loading (pL) and temperature (Temp), as reported in [9] are given in column 1 of Table 1. Out of these 20 sample run, four samples selected in random are (4, 300, 8), (5, 250, 4), (4, 200, 8) and (6, 300, 8) with corresponding activity values being 56.95, 54.5, 55.43 and 48.67 respectively. The sample square matrix is

(23) $$M=\left(\begin{array}{cccc} 4 & 300 & 8 & 1\\ 5 & 250 & 4 & 1\\ 4 & 200 & 8 & 1\\ 6 & 300 & 8 & 1 \end{array}\right)$$

and det(*M*) = 800 which is non‐singular.

$$M_{1}=\left(\begin{array}{cccc} pH & pL & Temp & 1\\ 5 & 250 & 4 & 1\\ 4 & 200 & 8 & 1\\ 6 & 300 & 8 & 1 \end{array}\right),$$

$$M_{2}=\left(\begin{array}{cccc} 4 & 300 & 8 & 1\\ pH & pL & Temp & 1\\ 4 & 200 & 8 & 1\\ 6 & 300 & 8 & 1 \end{array}\right),$$

$$M_{3}=\left(\begin{array}{cccc} 4 & 300 & 8 & 1\\ 5 & 250 & 4 & 1\\ pH & pL & Temp & 1\\ 6 & 300 & 8 & 1 \end{array}\right),$$

$$M_{4}=\left(\begin{array}{cccc} 4 & 300 & 8 & 1\\ 5 & 250 & 4 & 1\\ 4 & 200 & 8 & 1\\ pH & pL & Temp & 1 \end{array}\right)$$

The determinant of the matrices *M*_1_, *M*_2_, *M*_3_ and *M*_4_ is

(24) $$\begin{array}{ccc} det\left(M_{1}\right) & = & 8pL-400pH\\ det\left(M_{2}\right) & = & 1600-200Temp\\ det\left(M_{3}\right) & = & 100Temp-8pL+1600\\ det\left(M_{4}\right) & = & 400pH+100Temp-2400 \end{array}$$

Using Equation (6), a unique linear polynomial derived by the MLIP method is

(25) $$f=\frac{19pL}{1250}-\frac{207pH}{50}-\frac{49Temp}{80}+\frac{1477}{20}$$

Using this polynomial in Equation (25), expected frequency is obtained for the remaining 16 runs mentioned in Table 1.

**TABLE 1:** Observed and expected temperature frequency for single polynomial computation

S. No	pH, pL Temp	Original Values (*O*_*i*_)	Linear Poly. MLIP	Second order Polynomial ‐MLIP method	Second order Polynomial ‐Method of MLS
			(*E*_*i*_)	(*E*_*i*_)	(*E*_*i*_)
1	(4, 300, 8)	56.95	56.96	59.825	59.82499999
2	(5, 250, 4)	54.89	54.50	54.890	54.87999999
3	(5, 250, 0)	53.56	56.95	51.990	51.98999999
4	(5, 250, 8)	52.87	52.05	52.870	52.85999999
5	(5, 250, 4)	54.39	54.50	54.890	54.88999990
6	(6, 200, 8)	48.20	47.15	48.200	48.19999999
7	(4, 200, 4)	56.56	57.88	56.560	56.54999990
8	(5, 250, 4)	54.67	54.50	54.890	54.88999999
9	(5, 250, 4)	54.50	54.51	54.890	54.88999999
10	(6, 300, 0)	49.98	53.57	49.980	49.97999990
11	(4, 300, 8)	57.45	56.95	59.825	59.82499999
12	(6, 250, 4)	51.95	50.36	51.950	51.93999999
13	(4, 200, 8)	55.43	55.42	55.635	55.63499999
14	(5, 250, 4)	54.78	54.50	54.890	54.88999999
15	(4, 250, 4)	59.30	58.64	59.300	59.29999990
16	(6, 300, 8)	48.67	48.66	48.670	48.66599990
17	(5, 300, 4)	54.34	55.26	55.655	55.65499999
18	(5, 200, 4)	53.08	53.74	53.080	53.07999990
19	(5, 250, 4)	54.74	54.50	54.890	54.88999999
20	(6, 200, 0)	49.02	52.05	49.020	49.01899990

#### 6.1.5 MLIP method for second order polynomials

A method for computing linear polynomial is extended for deriving second‐order polynomials by MLIP method. For three independent variables, this approach involves a maximum of nine variables with a constant: 1, pH, pL, Temp, (pH × pL), (pL × Temp), (pH × Temp), (pH)^2^, (pL)^2^, (Temp)^2^ having the sample square matrix order 10. Therefore, consider a set of data for deriving the second‐order polynomial, a randomly selected samples of 10 runs as (5, 250, 4), (5, 250, 8), (6, 200, 8), (4, 200, 4), (6, 300, 0), (6, 250, 4), (4, 250, 4), (6, 300, 8), (5, 200, 4) and (6, 200, 0) and their corresponding experimental outputs being 54.89, 52.87, 48.2, 56.56, 49.98, 51.95, 59.3, 48.67, 53.08 and 49.02, respectively. The derived second‐order polynomial is given by

(26) $$\begin{array}{ccc} f\left(pH,pL,Temp\right)\\ \;=\;40.51500-\left(5.40250\times pH\right)+\left(0.22570\times pL\right)\\ \;+\left(2.7087500\times Temp\right)\\ \;-\left(0.01860\times pH\times pL\right)-\left(0.00061250\times pL\times Temp\right)\\ \;-\left(0.24312500\times pH\times Temp\right)+0.7350\times {\left(pH\right)}^{2}\\ \;-0.0002090\times {\left(pL\right)}^{2}\\ & & -0.153750\times {\left(Temp\right)}^{2} \end{array}$$

#### 6.1.6 Method of MLS for second order polynomials

From Table 1, temperature variable is zero and therefore the determinant of sample square matrices are singular. In this case, the MLIP method is not applicable (Refer Section 6.1.4). Therefore, the method of MLS is preferred to derive a second‐order polynomial. For this approach, the sample runs are assumed from the above MLIP method having maximum of nine variables involved in the polynomial which includes constant. Using Equations (20) and (22), the Vandermonde matrix obtained using three independent variables for second‐order polynomial is given by

(27) $$\left(\begin{array}{cccccccccc} 1 & 5 & 250 & 4 & 1250 & 1000 & 20 & 25 & 62500 & 16\\ 1 & 5 & 250 & 8 & 1250 & 2000 & 40 & 25 & 62500 & 64\\ 1 & 6 & 200 & 8 & 1200 & 1600 & 48 & 36 & 40000 & 64\\ 1 & 4 & 200 & 4 & 800 & 800 & 16 & 16 & 40000 & 16\\ 1 & 6 & 300 & 0 & 1800 & 0 & 0 & 36 & 90000 & 0\\ 1 & 6 & 250 & 4 & 1500 & 1000 & 24 & 36 & 62500 & 16\\ 1 & 4 & 250 & 4 & 1000 & 1000 & 16 & 16 & 62500 & 16\\ 1 & 6 & 300 & 8 & 1800 & 2400 & 48 & 36 & 90000 & 64\\ 1 & 5 & 200 & 4 & 1000 & 800 & 20 & 25 & 40000 & 16\\ 1 & 6 & 200 & 0 & 1200 & 0 & 0 & 36 & 40000 & 0 \end{array}\right)\left(\begin{array}{c} a^{\prime }\\ b^{\prime }\\ c^{\prime }\\ d^{\prime }\\ e^{\prime }\\ f^{\prime }\\ g^{\prime }\\ h^{\prime }\\ k^{\prime }\\ m^{\prime } \end{array}\right)\;=\;\left(\begin{array}{c} 54.59\\ 52.87\\ 48.20\\ 56.56\\ 49.98\\ 51.95\\ 59.30\\ 48.67\\ 53.08\\ 49.02 \end{array}\right)$$

Thus, using Equation (22), the second‐order polynomial is

(28) $$\begin{array}{c} f\left(pH,pL,Temp\right)\\ \;=\;40.51500000109080-\left(5.40250057412231\times pH\right).\\ \;+\;\left(0.22569998099311\times pL\right)\\ \;+\;\left(2.70875044599233\times Temp\right)\\ \;-\;\left(0.01860000318557\times pH\times pL\right)\\ \;-\;\left(0.00061250007178\times pL\times Temp\right)\\ \;-\;\left(0.24312500483848\times pH\times Temp\right)\\ \;+\;0.73499991762037\times {\left(pH\right)}^{2}\\ \;-\;0.00020900000045\times {\left(pL\right)}^{2}\\ \;-\;0.15374999999995\times {\left(Temp\right)}^{2} \end{array}$$

We observe that, the second‐order polynomial fit by both mathematical methods are closer and the maximum activity values are depicted in Table 1.

#### 6.1.7 Chi‐square testing for goodness of fit

To validate the above all polynomials, the *χ* ^2^ testing for goodness of fit is applied which consists of the following five steps:

Step 1 *Null hypothesis* *H*_0_: “*The multivariate polynomial fit is good one*”. That is, there is no significant difference between the observed and expected frequencies. Hence, *H*_0_: $\sum_{i=1}^{k}O_{i}=\sum_{i=1}^{k}E_{i}$.

The observed frequency as the original experimental values and its corresponding expected frequency is the simulated outputs calculated from the linear MLIP polynomial using Equation (25), and second order polynomials using Equations (26) and (28) respectively.

Step 2 *Alternative hypothesis* *H*_1_: ‘*The multivariate polynomial fit is not good one*’. That is, there is a significant difference between the observed and expected frequencies. Hence, $H_{1}:\sum_{i=1}^{k}O_{i}\neq \sum_{i=1}^{k}E_{i}$.

Step 3 Under *H*_0_, the *χ* ^2^ test statistic is:

$$\begin{array}{cc} \;\chi^{2}=\overset{k}{\underset{i=1}{\sum }}\frac{{\left(O_{i}-E_{i}\right)}^{2}}{E_{i}}∼\left(k-1\right)\;d.f=0.000007449600\;\;\;\;\;\left(for\;linear\;polynomial\;by\;MLIP\right)=0.000017738610\;\;\;\;\;\left(for\;2^{\text{nd}}\;order\;polynomial\;by\;MLIP\right)\\ & =0.000007756862\;\;\;\;\;\left(for\;2^{\text{nd}}\;order\;polynomial\;by\;MLS\right) \end{array}$$

From the above test statistic, we observe that there is a negligible error difference between estimated and actual values showing good inferences about the polynomials.

Step 4 The table value of *χ* ^2^ distribution for 3 degree of freedom to fit linear polynomial by MLIP method at *α* = 0.05 level of significance is $\chi_{\alpha }^{2}$ = 7.815. For both the MLIP and MLS methods, the table for fitting of second‐order polynomials at 9 degree of freedom with *α* = 0.05 level of significance is 16.919.

Step 5 The data yields a value for the Chi‐squared test statistic (calculated value) which does not exceed theoretical value for both polynomial methods. Therefore, *H*_0_ is accepted.

**FIGURE 5:**
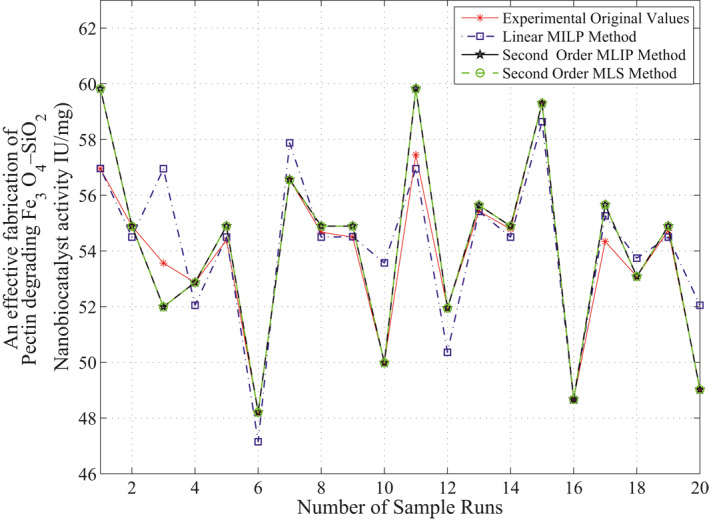
Chi‐square testing for goodness of fit for single sample

The observed and expected frequencies for linear and second‐order polynomials are shown in Table 1 and the comparisons are graphically shown in Figure 5. It is inferred from Figure 5 that there is no significant difference between the observed and expected frequencies obtained by methods. Hence, it may be concluded that ‘*the multivariate polynomial is a good fit*’.

In this small sample test, polynomials are derived using only one sample square matrix as four out of 20 sample runs using MLIP method and 10 out of 20 sample runs using MLS method with their polynomial validation is accepted.

#### 6.1.8 Normal distribution curve fit

Increases in polynomial validation follows large sample test. The hypothesis of the problem is similar to small sample test. The normal distribution curve can be drawn by experimental values versus the simulated outputs estimated by the polynomials.

**FIGURE 6:**
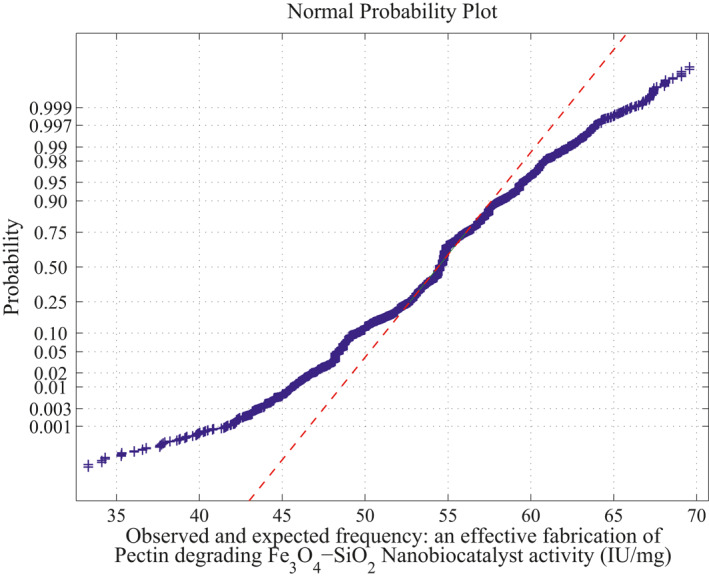
Linear polynomial: normal curve fit for 43,400 probability values

**FIGURE 7:**
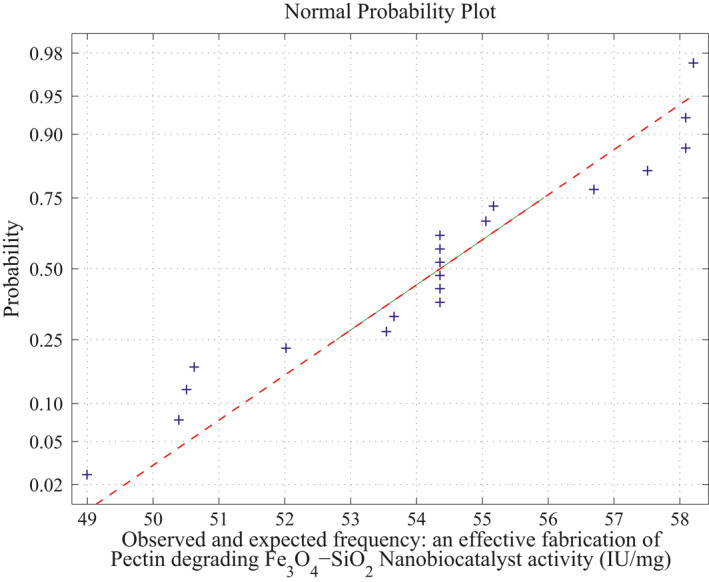
Linear polynomial: average normal plot for Figure 6

**FIGURE 8:**
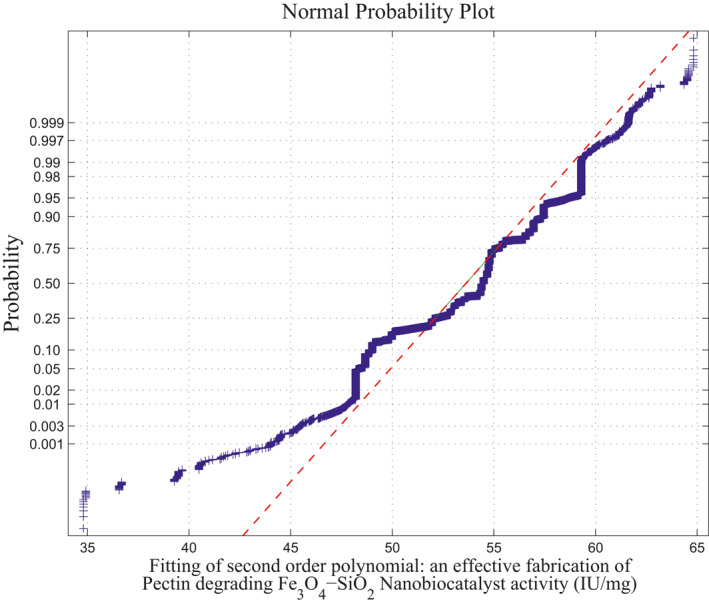
Second‐order polynomial: normal curve fit for 59,380 probability values

**FIGURE 9:**
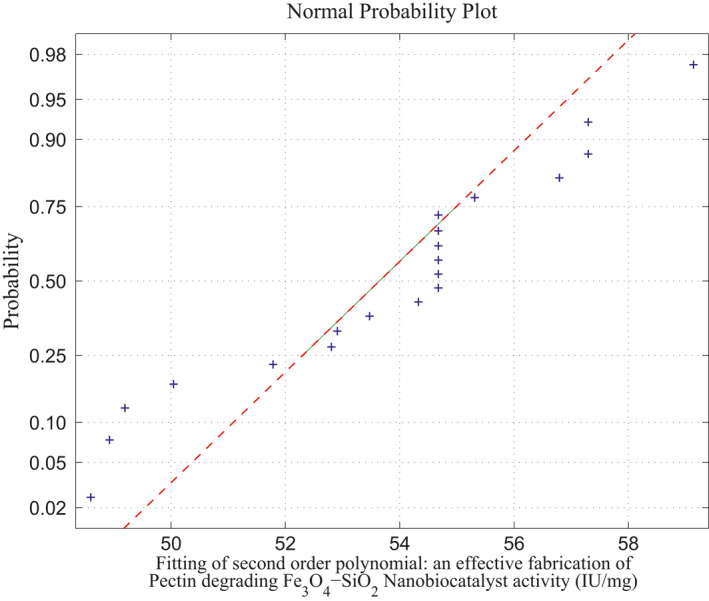
Second‐order polynomial: average normal plot for Figure 8

**FIGURE 10:**
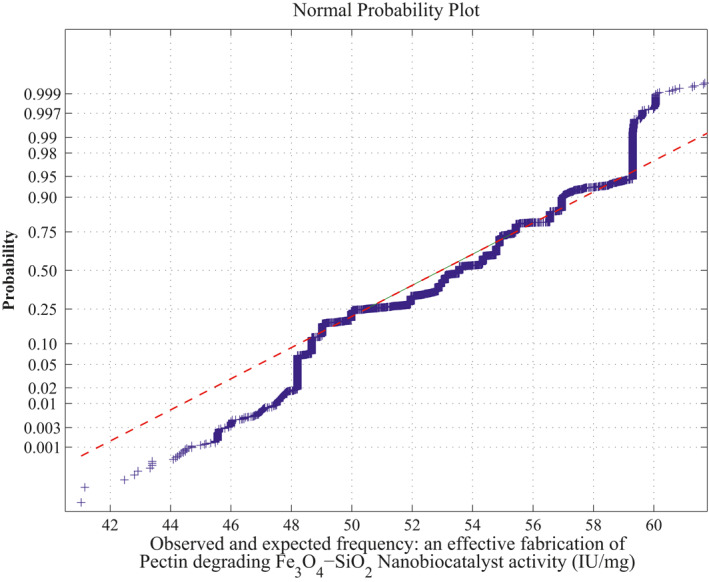
Second‐order polynomial: normal curve fit for 20,085 probability values

**FIGURE 11:**
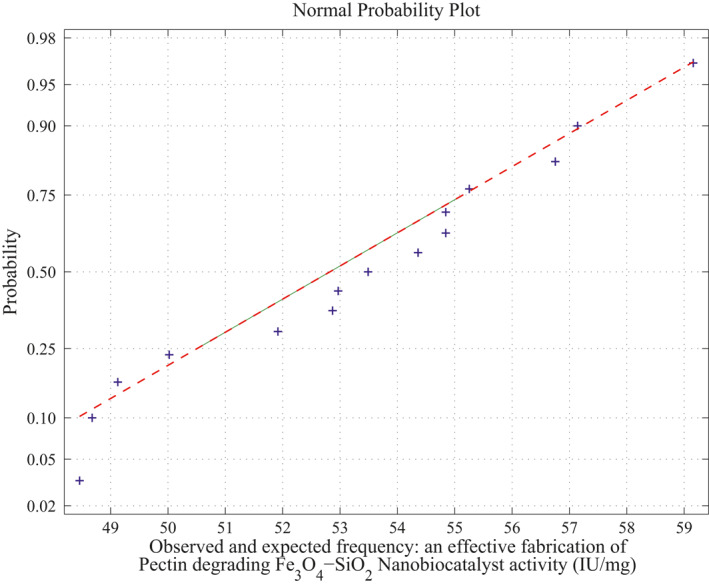
Second‐order polynomial: average normal plot for Figure 10

1. *Method of MLIP for Linear Polynomial:* There are 20 sample runs over three independent variables as inputs which adopt only maximum four sample runs for simulating each polynomial computation. Therefore, totally, 20*C*_4_ combinations of sample runs gives 2170 linear polynomials in which the sample square matrices are non‐singular out of 4845 combinations and remaining 2676 matrices are singular (ill‐conditioned). Hence, the linear polynomials evaluated using these samples run to fit a normal curve. The approximation of normal curve fit and its average probability plots are taken from 2170 polynomials per each sample matrix are shown in Figure 6 and Figure 7. The mean and standard deviation of the distribution is 54.2341 and 2.6348 respectively. Also, estimates of 95% confidence interval for mean lie between 54.209312 and 54.258888.
2. *Method of MLIP for Second‐Order Polynomial:* The above approach is extended into the second‐order polynomial computations. Three independent variables involved to derive a second‐order polynomial over maximum of 20*C*_10_ combination of samples runs. There are 2969 second‐order polynomials derived out of 184,756 square matrices, and the remaining 181,787 sample square matrices are singular (avoid ill‐condition, approximately 10^−7^ precision is considered for determinant value). Using the above procedure, the fitting of normal curve has mean 53.7983 and standard deviation is 2.8391. Moreover, estimates 95% confidence interval for mean is: 54.209312 and 54.258888. The normal probability plot and its average normal probability plots are shown in Figure 8 and Figure 9.
3. *Method of MLS for Second‐Order Polynomial:* Fitting of normal curve is also similar approach of MLIP method. This method adopts only second‐order polynomials over 20*C*_10_ combinations of sample runs. Specifically, the computation for inverse of a matrix has too high precision while the value of determinant is too small. Selection of sample matrices is reduced by repetitions of sample inputs out of 20 runs as referred in Table 1. In this table, the repetitions of 5^th^, 8^th^, 9^th^ and 19^th^ sample runs were the same as second run, also the 11^th^ run was same as first run. Apart these repetition runs, 15 sample runs were used for computation out of 20 runs to minimize the size of the sample runs which gives less number of sample square matrices. Totally, about 1339 polynomials were derived out of 3003 (minimizes from 184,756 count) sample square matrices for the computations, while by avoiding, 1664 matrices were singular (avoid ill‐condition, and approximately 10^−7^ precision is considered for the determinant value). Moreover, the normal distribution having mean and standard deviation is: 53.3259 and 3.1192. In addition to this, estimates having 95% confidence limit for mean lie between 53.2828 and 53.3690. The normal curve plot and its average probability plot are shown in Figures 10 and 11.

In this study, all probability plots having the simulated outputs is very closer to the experimental values and inference of testing the hypothesis is accepted.

#### 6.1.9 Comparison of results

The mathematical results are compared to experimental study in [9], as the empirical polynomials fitted by both methods shows significance results. The maximum activity of Fe_3_O_4_‐SiO_2_ Nanobiocatalyst obtained using linear MLIP method is: 58.64 with pH = 4, pL = 250 and Temp = 4°C. In the second‐order polynomials, for pH = 4, pL = 300 and Temp = 8°C have a maximal activity by the MLIP method which is 59.825 and by the method of MLS is 59.8249 as depicted in Table 1. For polynomial fitting, the simulation conducted for different sample runs is being follows large sample test. Hence, the statistical validation of each empirical polynomial is closely fitted to the experimental data in order to determine the effect of relationship between variables. Thus, the inference of statistical validation shows that the *fitting of multivariate polynomial is good one*. In experiment, the maximum activity was at 54.39 IU/mg when pH = 4.0; Temp = 4°C and pL = 250 *μ*g while the canonical analysis was performed for the successful optimization for fabrication of nanobiocatalyst. The glutaraldehyde concentration was fixed as 10% and the independent variables are optimized using OFAT method followed by RSM.

### 6.2 Application 2: polynomial fit for passive DHR system in NPP

The studies of this application is under NPP and the detailed experiments are discussed in the following which further leads mathematical analysis.

#### 6.2.1 The DHR problem in NPP

The objective of the problem is to evaluate the reliability of the passive DHR system. The success criteria of the system is that the fuel clad and the structural temperatures viz., Hot Pool Temperature (HPT), Central Sub Assembly Clad Hot Spot Temperature (CSACHST) and Storage Sub Assembly Clad Hot Spot Temperature (SSACHST) do not exceed their respective design safety limits such as 650°C, 1200°C and 950°C respectively. Moreover, in this problem the HPT, CSACHST and SSACHST are considered as dependent variables. Since the execution of the software code to evaluate the temperatures is time consuming, to estimate the reliability of the system using a representative equation, MLIP method is adopted. Using this method, to verify the optimal temperature do not exceed design safety limits. About 50 experimental runs are performed for constructing the polynomial. Fourteen significant parameters used for evaluating the reliability of the system are identified as: *K*_*Pre*_, *K*_*Dhxp*_, *K*_*Ic*_, *K*_*Air*_, *h*_*Dhx*_, *h*_*Ahx*_, *A*_*Dhx*_, *A*_*Ahx*_, *T*_*Air*_, *Pre*, *T*_*p*_, *T*_*s*_, *T*_*n*_, *T*_*d*_ the details of which are explained in [20]. The sample runs are taken from the experimental values is more useful for further mathematical computations while the sample square matrix must be non‐singular. The detailed polynomial computations are discussed in the following.

#### 6.2.2 MLIP method for Linear Polynomial Computation

The MLIP method is applied to this problem similar to the previous application. The hypothesis is to test the relation between observed frequencies of three dependent variables HPT, CSACHST and SSACHST against the simulated outputs as the expected frequency of the output response from MLIP method. Therefore, to derive the temperature frequency of output responses: *f*(*HPT*), *f*(*CSACHST*), *f*(*SSACHST*) for dependent variables are represented in equations from (29) to (31) given by

(29) $$\begin{array}{l} f\left(HPT\right)=77.782219819632\;K_{PRE}\\ \;-\;42.096645476289\;K_{DHXP}-31.745873183089\;K_{IC}\\ \;+\;186.989665308884\;K_{AIR}-294.913931066576\;h_{DHX}\\ \;-\;107.225829440823\;h_{AHX}+4.420951347223A_{DHX}\\ \;+\;31.825060886859\;A_{AHX}-125.920533158742\;T_{AIR}\\ \;-\;81.852645440360\;Pre+73.486043089831\;T_{n}\\ \;+\;17.566786039489\;T_{d}+24.440443560754\;T_{P}\\ \;+\;60.586037245240\;T_{S}-3302.419534352170 \end{array}$$

(30) $$\begin{array}{l} f\left(CSACHST\right)=-\;249.1951971201760\;K_{PRE}\\ \;+\;139.2890855498670\;K_{DHXP}\\ \;-\;279.0703797916200\;K_{IC}-192.6072917411010\;K_{AIR}\\ \;+\;65.3959560743662\;h_{DHX}+232.5183279357910\;h_{AHX}\\ \;-\;16.2464507730983\;A_{DHX}+1.4730836130343\;A_{AHX}\\ \;+\;17.0884264854431\;T_{AIR}-61.4473758399934\;Pre\\ \;+\;16.5178690283433\;T_{n}-102.5551707364020\;T_{d}\\ \;+\;283.1403081252710\;T_{P}+4.4711305701670\;T_{S}\\ \;+\;2799.642766849790 \end{array}$$

(31) $$\begin{array}{c} f\left(SSACHST\right)=-109.4098923029040\;K_{PRE}\\ \;-\;16.7118984123392\;K_{DHXP}\\ \;-\;41.5629331867813\;K_{IC}+180.1420798369090\;K_{AIR}\\ \;-\;187.5149250660080\;h_{DHX}+151.0601459602000\;h_{AHX}\\ \;+\;54.8655417234219\;A_{DHX}+15.1867302711122\;A_{AHX}\\ \;+\;20.5116521154908\;T_{AIR}+122.8154196920770\;Pre\\ \;-\;28.8866263797481\;T_{n}+132.8818692114280\;T_{d}\\ \;-\;76.9480498146180\;T_{P}-49.3978005066472\;T_{S}\\ \;-\;6968.2818275358700 \end{array}$$

In Table 2, the observed frequencies obtained from the actual computational runs and expected frequencies derived using above polynomials are given.

**TABLE 2:** observed and expected temperature frequency: HPT, CSACHST and SACHST for single polynomial

S. No	Original Values (HPT)	Exp. Values (HPT)	Original (CSA‐CHST)	Exp. Value (CSA‐CHST)	Original (SSA‐CHST)	Exp. Value (SSA‐CHST)
1	649.6	675.07362	1085.6	1157.47997	814.3	797.57605
2	643.8	643.75999	1094.6	1094.58999	789.3	789.28998
3	634.4	643.21452	1068.4	1083.71300	781.5	793.23825
4	646.3	646.29000	1072.7	1072.68991	821.2	821.15998
5	635.2	624.01837	1078.4	1050.83620	794.8	753.94578
6	633.1	633.29996	1073.6	1073.58999	780.9	780.82998
7	642.1	616.10978	1085.1	1114.96405	784.4	844.41758
8	624.6	632.67727	1114.8	1146.62578	792.1	821.29099
9	629.2	667.35138	1083.8	1089.10357	786.3	789.37526
10	628.7	628.69960	1054.2	1054.19299	780.7	780.67998
11	638.9	624.06136	1072.3	1077.18682	805.3	796.12761
12	640.9	657.86159	1097.8	1111.80216	784.6	820.81908
13	637.8	596.98909	1097.6	1081.50013	797.9	795.88379
14	638.9	638.88995	1089	1088.98999	802.7	802.69798
15	634.6	619.04429	1079	1142.79858	804.9	797.92517
16	633.5	673.86181	1096.6	1092.35269	793.3	758.27552
17	639.0	638.99700	1064.7	1064.67999	803	802.98998
18	640.6	643.28923	1075.7	1112.70293	796.9	830.59095
19	635.8	617.90108	1044.8	1042.24805	820.1	782.15588
20	636.4	636.38000	1037.7	1037.69991	806.7	806.69798
21	630.2	590.48484	1096	1101.37691	813.6	793.76299
22	630.3	661.37687	1072.2	1133.50869	783	766.70721
23	640.0	639.99300	1024.5	1024.47998	836.8	836.78998
24	633.3	615.41134	1058.5	1133.20233	795.8	796.21408
25	638.2	575.78188	1028.3	1082.91289	801.5	733.96968
26	638.3	638.29900	1100.6	1100.57995	792.7	792.68998
27	637.2	667.73988	1083.7	1066.70723	807.4	816.48616
28	618.6	678.51583	1084.1	1070.56832	775.8	872.92135
29	632.7	632.69500	1057.6	1057.58998	777.3	777.26998
30	637.1	625.23807	1077.2	1164.00382	816.5	782.35711
31	643.7	625.31415	1059.6	1088.56706	816	850.89798
32	637.3	651.08291	1069.9	1133.92527	811.1	817.89423
33	629.4	629.36000	1062.5	1062.47991	783.8	783.79598
34	639.0	655.01785	1074	1142.00408	790.2	835.89488
35	636.5	674.71562	1092.2	1086.50945	791.9	795.76720
36	647.3	647.29999	1042.3	1042.29990	812.9	812.89798
37	640.4	670.26116	1103	1054.77981	813.5	757.25437
38	651.9	607.35248	1073.7	1085.50608	817.6	812.61949
39	645.5	645.49994	1069.3	1069.29997	808.1	808.09998
40	644.5	652.04562	1025.1	1072.22319	825.9	910.20027
41	639.4	574.60474	1073.1	1044.58695	816.8	860.03078
42	649.6	649.59998	1088.9	1088.89996	831.3	831.29999
43	637.2	606.70144	1117.1	1090.45455	781.6	800.72866
44	629.0	628.41481	1072.1	1038.71618	783.2	748.81006
45	629.1	634.70355	1087	1037.29209	787.2	821.66816
46	635.5	635.49989	1014.3	1014.29989	794.6	794.59999
47	629.6	603.50246	1038.8	1046.88555	786.1	825.54927

#### 6.2.3 MLIP & method of MLS for second order polynomials

From Section 6.2.2, the derivation of second‐order polynomial is computationally not possible, since the sample square matrix consists of all possible second‐order terms for 14 independent variables. Moreover, order of the matrix is too large (order 120× 120) with determinant is very low, in this case the inverse matrix computation is too high (ill‐condition). Thus, the higher order polynomials derived by both methods are not possible to attain good results.

#### 6.2.4 Chi‐square testing for goodness of fit

To validate the linear polynomials, *χ* ^2^ testing for goodness of fit for three dependent variables are discussed in the following.

*Step 1: Null hypothesis* *H*_0_:

1. *The multivariate polynomial fit for* HPT *is a good one*,
2. *The multivariate polynomial fit for* CSACHST *is a good one*,
3. *The multivariate polynomial fit for* SSACHST *is a good one*.

that is, there is no significant difference between the observed and expected frequencies for HPT, CSACHST, SSACHST.

*H*_0_: $\overset{k}{\underset{i=1}{\sum }}O_{i}=\overset{k}{\underset{i=1}{\sum }}E_{i}$

Notice that, the observed frequency are the original experimental values depicted in Table 2 and its corresponding simulated output as the expected frequency as the simulated outputs are computed from Equations (29) to (31).

*Step 2: Alternative hypothesis* *H*_1_: *The multivariate polynomial fit is not good one* for three dependent variables. That is, there is significant difference between the observed and expected frequencies.

$H_{1}:\overset{k}{\underset{i=1}{\sum }}O_{i}\neq \overset{k}{\underset{i=1}{\sum }}E_{i}$

*Step 3:* Under *H*_0_, the *χ* ^2^ test statistic is:

$$\begin{array}{ccc} \chi^{2}\; & = & \;\overset{k}{\underset{i=1}{\sum }}\frac{{\left(O_{i}-E_{i}\right)}^{2}}{E_{i}}∼\left(k-1\right)\;d.f\\ & = & 6.92386E-05\;\left(MLI\;Plinear\;polynomial\;of\;HPT\right)\\ & = & 2.02581E-06\;\left(MLI\;Plinear\;polynomial\;of\;CSACHST\right)\\ & = & 1.04369E-05\;\left(MLI\;Plinear\;polynomial\;of\;SSACHST\right) \end{array}$$

From the above test statistic, we observe that the negligible error difference between estimated and actual values shows good inferences about the polynomials.

*Step 4:* The table value of *χ* ^2^ distribution for 14 degree of freedom for linear polynomials derived by MLIP method with *α* = 0.05 level of significance is $\chi_{\alpha }^{2}=23.685$. Here, the Chi‐square test statistic value does not exceed the theoretical values. Thus, there is no statistical evidence against the null hypothesis.

*Step 5:* The null hypothesis of the problem cannot be rejected. Therefore, the data yields the inference about ‘*the multivariate polynomial fit for* HPT, CSACHST *and* SSACHST *is good one*’.

The comparison between the observed and expected frequency temperature for the output response: HPT, CSACHST and SSACHST is depicted in Table 2.

There are only one sample square matrix is considered over 15 (randomly picked) out of 47 runs with their simulated output is closer to the original experimental values. Once a polynomial with a statistically good fit is obtained, the polynomial representation can subsequently be used for deriving the reliability estimate of the system by simulating the input parameters. Monte–Carlo simulation is a widely accepted and consistent method for simulation.

#### 6.2.5 Normal distribution curve fit

In this problem, an increase of polynomial validation follows a large sample test. The hypothesis of this problem is similar to small sample test discussed in Section 6.2.4.

**FIGURE 12:**
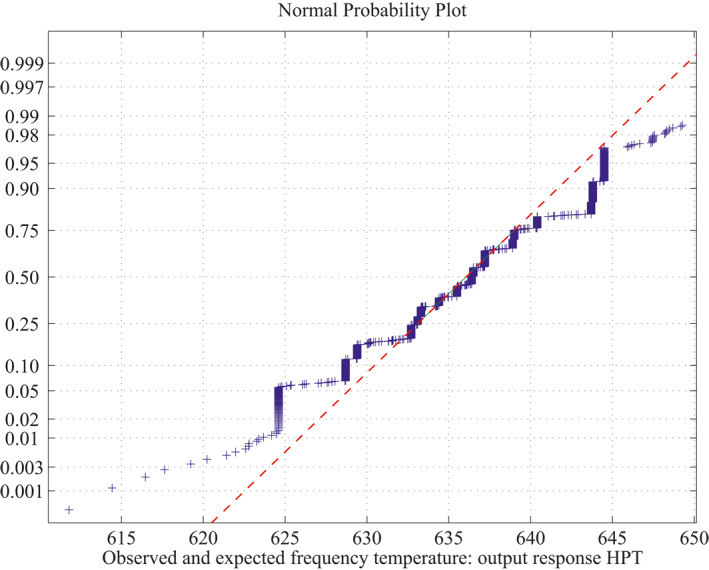
Linear polynomial: normal curve fit for 1314 probability values

**FIGURE 13:**
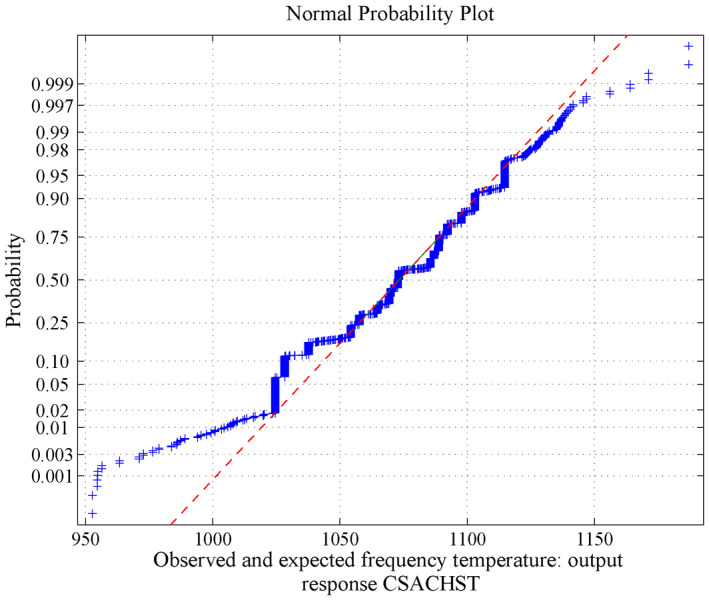
Linear polynomial: normal curve fit for 2196 probability values

**FIGURE 14:**
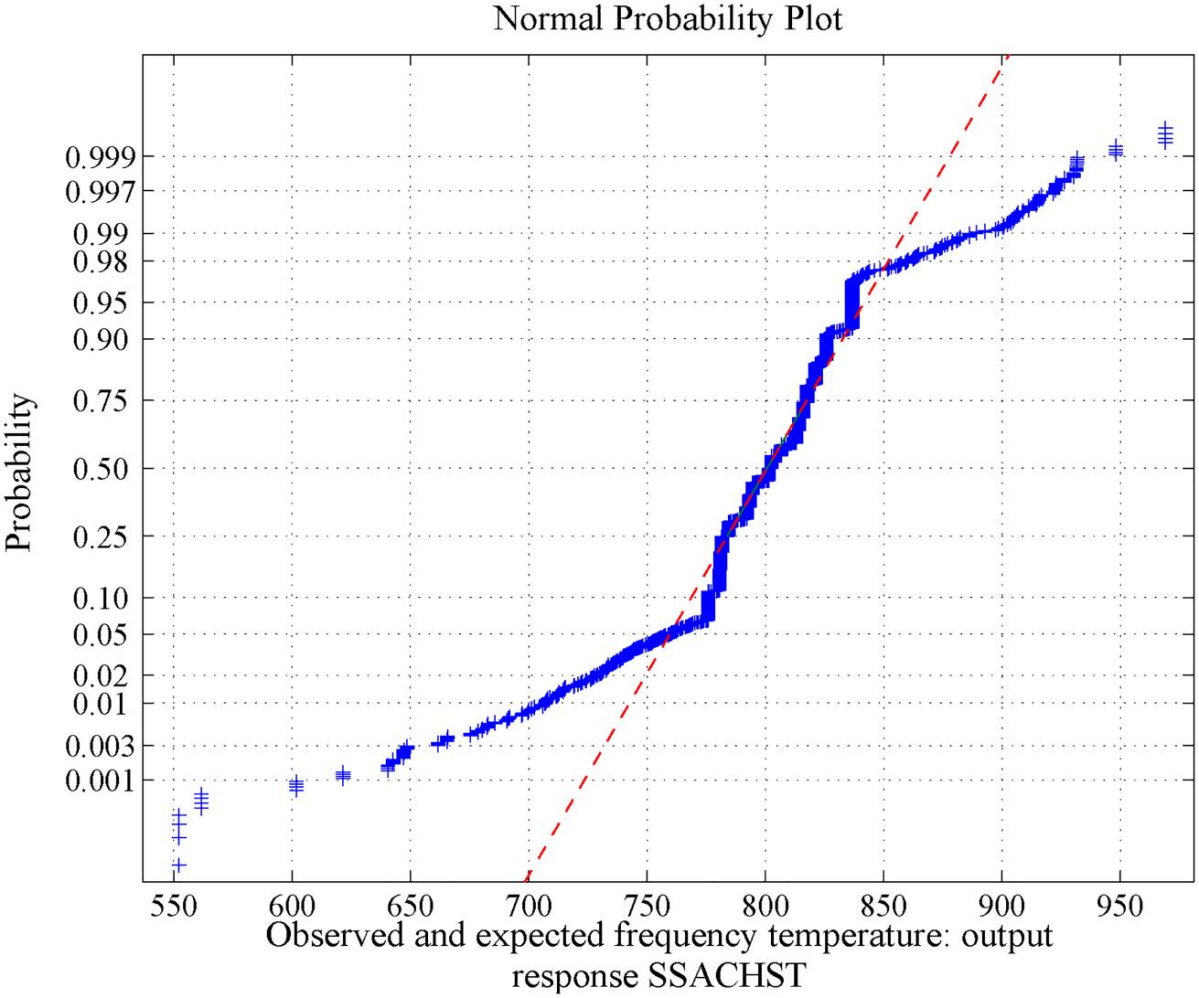
Linear polynomial: normal curve fit for 3340 probability values

**FIGURE 15:**
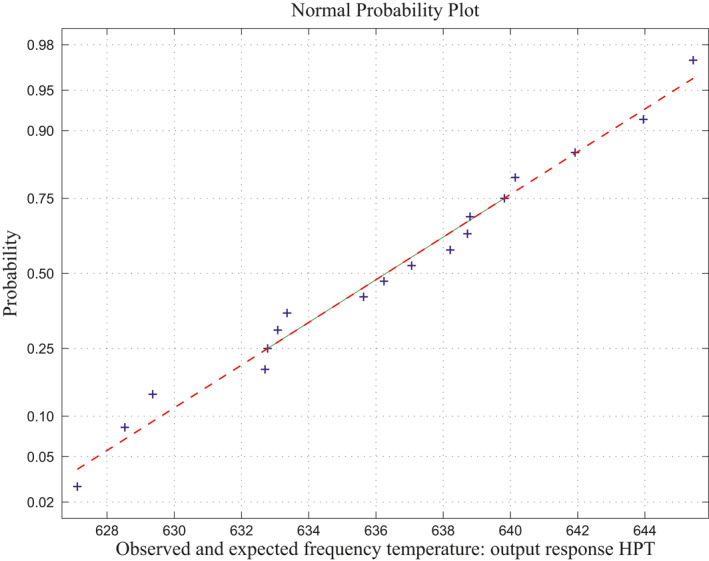
Linear polynomial: average normal plot for Figure 12

**FIGURE 16:**
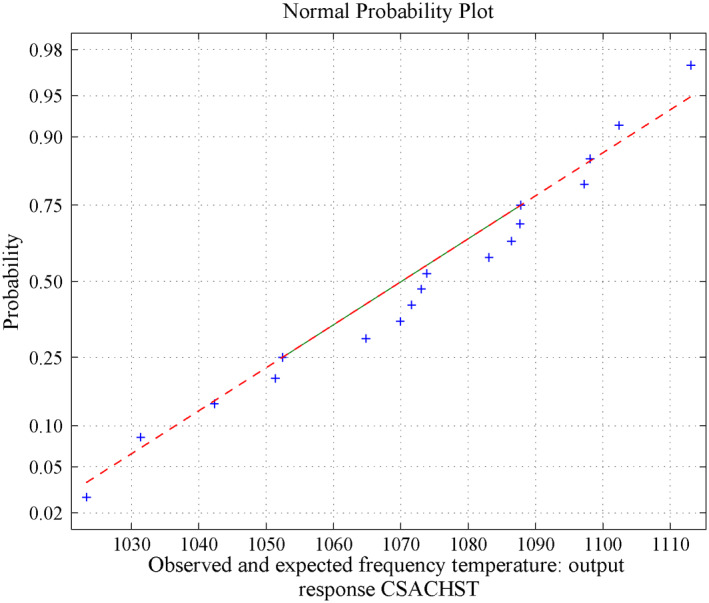
Linear polynomial: average normal plot for Figure 13

**FIGURE 17:**
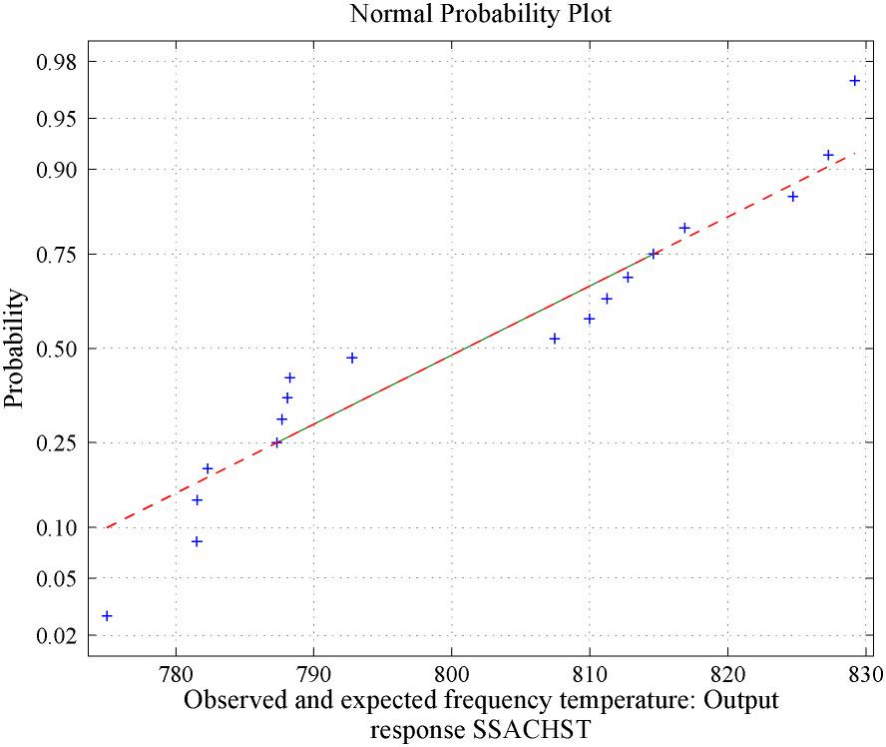
Linear polynomial: average normal plot for Figure 14

1. *MLIP method for Linear Polynomial Computation:* Procedure to fit a normal curve is discussed here. Using the MLIP method, the problem of having 14 independent over three dependent variables adopts a maximum of 15 sample runs for simulating each polynomial. For large sample test, total number of sample square matrices are 47*C*_15_ combination of sample runs which gives large number of matrices. Avoiding more time consumption, only maximum 18 sample runs were considered which are randomly picked out of 47 sample runs. Now, the MLIP method is applied only for 18*C*_15_ combinations of sample runs to compute the number of linear polynomials in which the matrix is non‐singular. Also, estimates of 95% confidence interval bounds and the characteristics (mean and standard deviation) of the normal curve are discussed below.
  (a) For 18 sample runs, 73 linear polynomials were derived for the dependent variable HPT and remaining 743 sample square matrices are closer to singular (avoid ill‐condition, upto 10^−7^ precision above is considered for non‐singular) out of 816 combinations. The mean and standard deviation are 636.2697 and 5.0281, and estimates of 95% confidence interval for the mean lie between 635.99783 and 636.54157.
  (b) For 18 sample runs, 122 linear polynomials were derived for the dependent variable CSACHST and the remaining 694 sample square matrices are closer to singular (avoid ill‐condition, upto 10^−7^ precision above is considered for non‐singular) out of 816 combinations. The mean and standard deviation is: 1072.8 and 24.1708, estimates 95% confidence interval for mean lie between 1071.789048 and 1073.810952.
  (c) For 18 sample runs, 167 linear polynomials were derived for the dependent variable SSACHST and remaining 649 sample square matrices are closer to singular (avoid ill‐condition, upto 10^−7^ precision above is considered for non‐singular) out of 816 combinations. For SSACHST, the mean and standard deviation is: 801.0357 and 17.3081, estimates 95% confidence interval for mean lie between 800.416955 and 801.654445.
    The normal curves for HPT, CSACHST and SSACHST are shown in Figure 12, Figure 13 and Figure 14, respectively. Furthermore, the average of normal probability plots of HPT, CSACHST and SSACHST for different polynomials per each sample run are shown in Figure 15, Figure 16 and Figure 17 respectively. In all probability plots, the three dependent variables satisfythe respective required design safety limits.

2. *MLIP and method of MLS for Second‐Order Polynomials:* For normal probability plots, the second‐order polynomial computation depends on the sample square matrix of order 120×120. According to Section 6.2.3, there are 18 runs out of 47 sample runs were picked randomly and determinant value for all matrices (18*C*_15_ combinations) are singular (ill‐condition). In all 816 combinations, the determinant values of the matrix using the MLIP method decreases, whereas the inverse of a matrix using the method of MLS increases. Hence, the fitting of second‐order polynomials are computationally not possible to provide good results.

In all probability plots, the simulated outputs are very closer to the experimental values and validation for testing the hypothesis is accepted for this problem.

#### 6.2.6 Comparative study

A set of 47 observed values obtained from the actual experimental runs performed in the system are compared with that of the polynomial estimates and it is seen from the chi‐square test that there is a negligible error in the estimated values compared to the experimental values. This has served as a comparative study and has provided the required confidence in the polynomial fit. Moreover, for the 14 parameters being studied for the analysis, the number of experimental runs is found to be statistically sufficient based on Wilk's criteria.

## 7 MATLAB COMPUTATIONS

From two case studies, the computations of multivariate polynomials derived by these mathematical approaches are done using MATLAB programming language. The validations of these two real‐time applications are statistically analysed which are depicted in Table 3, Table 4, Table 5, Table 6 and Table 7.

**TABLE 3:** Statistical analysis of the first application by MLIP method

S. no	Computations	Linear polynomial	Second‐order polynomial
1	Method	MLIP method	MLIP method
2	No. of samples	20*C*_4_	20*C*_10_
3	Total matrices	4845 samples	184,756
4	Singular	2676	181,787
5	Non‐singular	2170	2969 (above 10^−7^decimal places)
6	Total polynomials	2170	2969
7	*χ* ^2^ testing (*α* = 0.05)	Accepted (3 d.f)	Accepted (9 d.f)
8	Normal fit	Done (size = 43400)	Done (size = 59380)
9	Mean and Std. deviation	54.2341 & 2.6348	53.7983 & 2.8391
10	95% confidence interval	54.20931 & 54.25888	53.77546 & 53.82113

**TABLE 4:** Statistical analysis of the first application by the method of MLS

S. No	Computations	Second‐order polynomial	Description
1	Method	Method of MLS	Inverse exists
2	No. of samples	15*C*_10_	15 out of 20 runs
3	Total matrices	3003 samples	Combination
4	Singular	1664	Close to zero
5	Non‐singular	1339	Discrete loop values
			Out of 3003 samples
6	Total polynomials	1339	Second‐order polynomials
7	*χ* ^2^ testing (*α* = 0.05)	Accepted	(9 d.f)
8	Normal fit	Done	Done (size = 20085)
9	Mean & Std. deviation	53.3259 and 3.1192	Characteristics
10	95% confidence interval	53.2828 to 53.3690	Mean

**TABLE 5:** Statistical analysis for HPT of the second application

S. no	Computations	Linear polynomial	Descriptions
1	Method	MLIP method	Without inverse
2	Total runs	47 sample runs	18 runs used
3	No. of samples	18*C*_15_	816 samples
4	Total matrices	816	Combination
5	Singular	743	Closer to zero
6	Non‐singular	73	Above 10^−7^decimal places
7	Total polynomials	73	Linear polynomial
8	*χ* ^2^ testing (*α* = 0.05)	Accepted	14 d.f
9	Normal fit	Done	size = 1314
10	Mean & Std. deviation	636.2697 & 5.0281	Characteristics
11	95% confidence interval	635.99783 to 636.54157	Mean

**TABLE 6:** Statistical analysis for CSACHST of the second application

S. no	Computations	Linear polynomial	Descriptions
1	Method	MLIP method	Without inverse
2	Total runs	47 sample runs	18 runs used
3	No. of samples	18*C*_15_	816 samples
4	Total matrices	816	Combination
5	Singular	694	Closer to zero
6	Non‐singular	122	Above 10^−7^decimal places
7	Total polynomials	122	Linear polynomial
8	*χ* ^2^ testing (*α* = 0.05)	Accepted	14 d.f
9	Normal fit	Done	size = 2196
10	Mean & Std. deviation	1072.8 & 24.1708	Characteristics
11	95% confidence interval	1071.78905 to 1073.81095	Mean

**TABLE 7:** Statistical analysis for SSACHST of the second application

S. no	Computations	Linear polynomial	Descriptions
1	Method	MLIP method	Without inverse
2	Total runs	47 sample runs	18 runs used
3	No. of samples	18*C*_15_	816 samples
4	Total matrices	816	Combination
5	Singular	649	Closer to zero
6	Non‐singular	167	Above 10^−7^decimal places
7	Total polynomials	167	Linear polynomial
8	*χ* ^2^ testing (*α* = 0.05)	Accepted	14 d.f
9	Normal fit	Done	size = 3340
10	Mean & Std. deviation	801.0357 & 17.3081	Characteristics
11	95% confidence interval	800.41695 to 801.65445	Mean

## 8 SUMMARY OF RESULTS

An important computational observations with conclusions obtained from two real‐time applications are outlined in the following.

1. In the NPP problem, 14 experimental runs are used to derive MLIP method for the output response of critical temperatures HPT, CSACHST and SSACHST.
2. In chemical application, 4 out of 20 sample runs are randomly chosen to derive a linear polynomial by the MLIP method and 10 samples are chosen to fit a second‐order polynomial by both the MLS and MLIP methods.
3. For both applications, the *χ* ^2^ testing for goodness of fit is applied to test single polynomial and the results indicates the polynomial is a best fit.
4. It is therefore inferred that the polynomial fit can be used for simulating the results when the computational time for the actual execution of the code is expensive. The polynomial can also be used to build the response surface simulation.
5. Practically, in DHR application the number of variable is 14 over three dependent variables derives each linear polynomial. Therefore, this MLIP method fails for second degree polynomial count is *C*(14 + 2, 2) in which the sample square matrix consists of order 120 is singular.
6. According to Equation (22), the method of MLS fails for DHR application, since the matrix is singular.
7. The MLIP method fails, when the sample run are collinear positions.

## 9 CONCLUSIONS AND FUTURE WORKS

Herein, if the number of sample square matrices (only non‐singular matrices) are less than or equal to 30, it follows a small sample test. This is possible, if the number of variables involved in the application is same as the number of sample square matrices. Also, polynomials were derived for one and only one matrix in which *χ* ^2^ testing for goodness of fit is accepted. If the number of non‐singular matrices are more (*n* > 30), it follows large sample test. Otherwise, the sample runs are more as compared to the number of variables; hence, we prefer a combination formula for selection of matrices. In this case, the polynomial validation by a large sample test is applied and the size of the sample is decided by *n* = *number of polyomials* × *size of sample runs*.

Even though, if both method fails when the matrix is singular, in practical scenario the determinant value tends to zero then, the inverse computation is practically not possible. To avoid this situation, we fix the precision of the determinant values in which the matrix is to be considered as non‐singular. In DHR problem, this precision is considered as above 10^−7^ decimal places (avoid for ill‐condition). For the time‐consuming, consider a random selection of 18 samples runs for the temperature variables HPT, CSACHST and SSACHST respectively. Moreover, the added advantages are that the MLIP method is computationally easy as compared to the method of MLS. In the MLS method, the inverse operation determines the co‐efficient of second‐order polynomials. It means, a low precision affects the inverse operation ${\left(Van◦Van^{T}\right)}^{-1}$ as well as affects this inverse by the product of transpose matrix ${\left(Van◦Van^{T}\right)}^{-1}\times Van^{T}$ (Refer Equation (22)). Therefore, this is to decide if the adoptable precision values classify the matrix as either singular or non‐singular. Another disadvantage is that if the number of variables is increased, and the method of MLS is difficult. Practically, the MLIP method is preferred when the values of (*Van ◦ Van* ^*T*^) are too high which leads to affect the inverse and determinant operations of the MLS method. Thus, the MLIP method is more suitable and the MATLAB computation provides good precision for the applications.

In the chemical engineering problem, we have 20*C*_4_ combinations and 20*C*_10_ combinations of sample matrices were used to fit the linear and second‐order polynomials. The maximum activity of Fe_3_O_4_‐SiO_2_ nanobiocatalyst activity achieved using the linear MLIP method is: 58.64 with *pH* = 4, *pL* = 250 and *Temp* = 4. In addition, *pH* = 4, *pL* = 300 and *Temp* = 8 has maximal activity for the fitting of second‐order polynomials by the MLIP method is 59.825, whereas in the method of MLS it is 59.82499999 as depicted in Table 1. In the second problem, success criteria of the DHR system are that the fuel clad and the structural temperatures namely: HPT, CSACHST, SSACHST do not exceed their respective design safety limits as verified using multivariate linear polynomials representation over 14 design variables (independent) by MLIP method mentioned in equation from (29) to (31). Furthermore, the higher order polynomials are not suitable, since the number of variables in second order is large, while their values are closer to zero. Also, the derived polynomials are validated by *χ* ^2^ testing for goodness of fit not rejected at 5% significance level.

Summary of the study was calculated from the mean and standard deviation and estimates at 95% confidence interval bounds. Thus, the normal probability curve is plotted and the average is determined samplewise for different polynomials. In the first application, the normal probability curve and its average probability plots are shown in Figures 6, 7, 8, 9, 10, 11 respectively. Also, for second problem, the normal probability curve and its average probability plots are shown in Figures 11, 12, 13, 14, 15, 16 respectively.

In both methods, the number of independent variable is small then the second‐order polynomial computation by either using the MLIP or the method of MLS gives similar polynomials in Equations (26) and (28) as discussed in the first application. The variables take integer values from both methods which control the inverse operation. In the MLS method, the sample square matrix is obtained from partial derivatives (Vandermonde matrix of three variables), while in the MLIP method it is obtained from matrix‐based approach. The second‐order polynomial by both methods is closer by applying the direct MLS method that does not guarantee even if the values are very small or the determinant is close to zero. If the matrix values are too small, we cannot determine the inverse and its product of matrices. Thus, the matrix of these methods depends on the size: the matrix column as the number of variables with same size of sample runs constructs a square matrix. Different case studies were discussed for fitting the linear and second‐order polynomials over number of independent versus dependent variables. Thus, the MLIP method has less computation with less time consumption. For both applications, the MLIP method is a more adoptable easy computation for solving real‐time applications. As a future work, this approach is extended for the quantification of reliability in the second problem. The number of variables is greater than the number of sample runs (this matrix must be rectangular), then a new procedure is required for polynomial computations.
